# Functional insights into dispensable genes using genome-wide loss-of-function burden tests in Arabidopsis

**DOI:** 10.1093/plcell/koag087

**Published:** 2026-03-27

**Authors:** Kehan Zhao, Mariele Lensink, J Grey Monroe

**Affiliations:** Department of Plant Sciences, University of California Davis, Davis, CA 95616, USA; Department of Plant Sciences, University of California Davis, Davis, CA 95616, USA; Department of Plant Sciences, University of California Davis, Davis, CA 95616, USA

## Abstract

Not all genes are essential for plant survival. With the rise of pan-genomics, it is evident that certain genes can be lost without negatively affecting fitness. Naturally occurring loss-of-function (LoF) mutations provide a valuable perspective on gene dispensability, offering insights into deleterious and adaptive gene loss. In this study, we identified 91,751 naturally occurring LoF variants from publicly available Arabidopsis genome data. Our findings demonstrate that LoF-intolerant genes are enriched in essential biological functions and associated with specific histone marks linked to active transcription. In contrast, LoF-tolerant genes exhibit relaxed selective pressure and are enriched in functions related to pollen rejection and defense responses and can be used as a proxy for dispensable genes in the pan-genome. Using a random forest model trained on histone marks, we achieved moderate success in predicting gene LoF tolerance, with an area under the curve (AUC) of 0.718 in Arabidopsis and 0.767 in rice, and even across species. We also pioneered genome-wide LoF burden tests in Arabidopsis, collapsing independent LoF alleles into a single state to reduce allelic heterogeneity. By integrating LoF burden tests with transcriptomic data, we identified thousands of LoF-expression associations. Notably, this analysis accurately recapitulated the flowering time networks and identified *FRIGIDA* as a key regulator of flowering time genes. Furthermore, we found that collapsing alleles based on functional outcomes enhances association sensitivity. These results provide insight into gene dispensability and a framework for leveraging LoF mutations to study gene functions with improved association studies.

## Teaser

Genome-wide loss-of-function burden tests in Arabidopsis reveal functional insights into dispensable genes and demonstrate how collapsing variants by functional consequence in association studies can improve gene discovery.

## Introduction

Not all genes are essential for an organism's survival. In the age of pan-genomics, it is clear that many genes within a species' genome can be absent or lost. Moreover, gene loss can often be adaptive (Smith 1970; Olson 1999; Orr 2005; Albalat and Cañestro 2016; Murray 2020; Klim et al. 2024). When certain genes become dispensable, their loss can lead to advantageous traits under specific environmental conditions or selective pressures. For example, functional disruption of *CBF* genes, which encode transcription factors that regulate the expression of various cold-responsive genes, improves fitness in warmer climates in Arabidopsis (Monroe et al. 2016; Lee et al. 2024). In humans, individuals with a homozygous knockout for *C-C chemokine receptor type 5* (*CCR5*) are resistant to HIV-1 infection (Liu et al. 1996). A plethora of adaptive gene loss cases has also been reported in other plant, animal, and microorganism species (Spielmeyer et al. 2002; Wong et al. 2012; Kvitek and Sherlock 2013; Flannick et al. 2014; Claessens et al. 2017; Sharma et al. 2018; Gomez et al. 2019; Cortinovis et al. 2024; Klim et al. 2024; Liu et al. 2024; Zhang et al. 2025). Adaptive gene loss contributes to the dynamic nature of gene content evolution, where genomes are continuously shaped by gains, losses, and modifications of genes (Sánchez-Serna et al. 2025). Previously in human studies, researchers have pointed out that loss-of-function (LoF) variants are valuable for identifying promising drug targets and assessing the safety of gene inhibition (Minikel et al. 2020). Artificial gene knockouts in agriculture, like the knockout of *CYP79D1* and *CYP79D2* in cassava to reduce cyanide levels by 92% (Jørgensen et al. 2005), can accelerate plant breeding and crop improvement.

Studying gene function in the context of dispensability provides insight into which genes are essential for basic cellular processes, and which can tolerate LoF mutations. Genes that can withstand LoF are typically non-essential but may play significant roles in specialized or redundant functions. Investigating the functions of these dispensable genes can reveal their contributions to phenotypic diversity and adaptation. In traditional genetic research, the process started by identifying a phenotypic change, followed by discovering the gene responsible for it, often resulting in the identification of previously unidentified genes. However, genes that had little or no influence on commonly studied traits were largely overlooked. Non-essential genes, by their nature, often don’t have significant effects on observable phenotypes, thus are less studied and have poor functional annotations.

Genome-Wide Association Studies (GWAS) aim to identify genetic variants associated with specific traits by testing for allele-phenotype relationships across large populations (Manolio 2010; Uffelmann et al. 2021). This method aids gene discovery and functional studies by revealing how genes contribute to complex traits and diseases, offering insights into new gene targets. However, allelic heterogeneity poses a significant challenge in conventional GWAS. Allelic heterogeneity occurs when different alleles at a single locus or different loci contribute to the same trait, such as multiple independent LoF variants leading to disrupted gene function, complicating the identification of causal variants (Bergelson and Roux 2010). Over the past decades, the availability of Whole-Genome Sequencing (WGS) data has revolutionized genomic studies, and advances in predicting variant effects from gene sequences have significantly contributed to human and medical research (MacArthur et al. 2012; Brandes et al. 2023; Neville et al. 2024), paving the way for the exploration of genes by leveraging natural genetic variation. To study rare protein-coding variants and enhance the statistical power of their analysis, burden tests were introduced. This approach aggregates genetic variants, typically loss-of-function variants, within a gene to construct a “burden genotype,” which is subsequently tested gene-by-gene for associations with phenotypes. LoF burden tests have proven instrumental in gene discovery within human genomics (Backman et al. 2021; Koprulu et al. 2022; Ivarsdottir et al. 2024; Spence et al. 2026). Although conceptually similar to GWAS, they excel in prioritizing the identification of distinct genes (Spence et al. 2026), offering a complementary and powerful tool for genetic research.

Utilizing LoF alleles for genome-wide association studies offers several additional advantages. Traditional GWAS relies on linkage to genetic markers, which often identify broad genomic regions containing hundreds of candidate genes, necessitating additional work to pinpoint causal loci. By directly associating functional states of individual genes with phenotypes, LoF association testing provides a more targeted approach. Furthermore, LoF burden tests provide actionable insights, offering testable hypotheses for experimental validation.

In this study, we performed genome-wide LoF burden tests that mitigate allelic heterogeneity by collapsing independent LoF alleles to a single allelic state (LoF burden). We tested this approach with transcriptome data, present evidence that collapsing alleles based on their functional outcomes enhances the sensitivity of association testing and offer a framework for gene function discovery through LoF-expression associations.

## Results and discussion

### Pan-genome gene dispensability can be inferred from predicted LoF based on a single reference genome

In total, we called 91,751 naturally occurring variants that have putative LoF effects on genes from publicly available WGS data. This is higher than previous estimates (Xu et al. 2019) because we combined both small-scale mutations and structural variants (see *Materials and Methods*). We emphasize that these are putative loss-of-function similar to related analyses in humans (MacArthur et al. 2012; Emdin et al. 2018; Rausell et al. 2020; Beaumont et al. 2024), and that their true effects would require further experimental validation. The majority of those LoF alleles have a lower expression level compared to their functional counterparts, as expected (Figure S1). The variant calling process is based on a single reference genome, Columbia (Col-0). To avoid confusion, we note that gene dispensability refers to variation in gene presence/absence across the species pan-genome, while LoF tolerance refers to the degree to which genes in the Col-0 reference genome can withstand loss-of-function mutations as predicted in our computational models.

**Figure 1:**
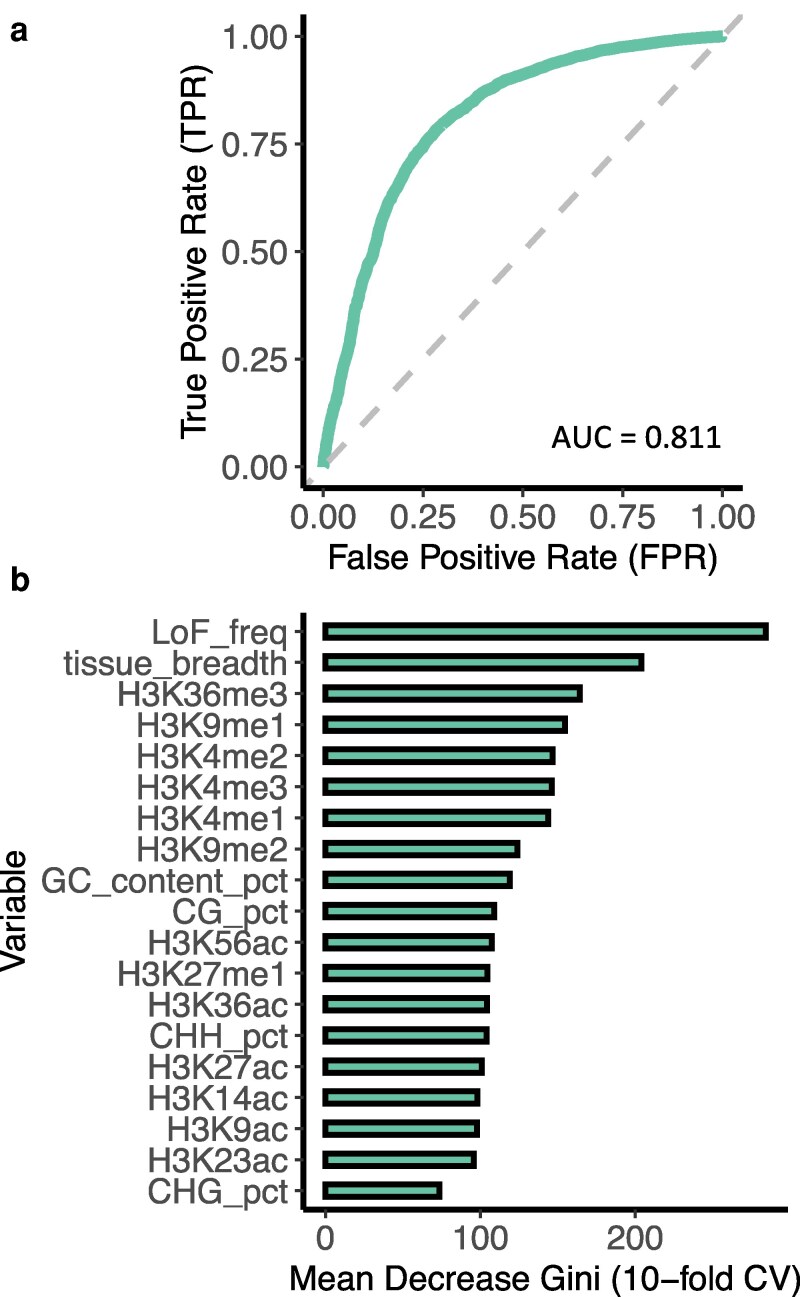
Random forest model of pan-genome gene dispensability prediction. **a)** The ROC curve of the predictive model using Random Forest (RF). An AUC-ROC value of ∼0.5 is equivalent to random guessing, while an AUC-ROC of 1 indicates perfect predictions. The diagonal dashed line shows the expected performance of a model based on random guessing. Curves closer to the upper left corner of the chart represent a better predictive performance than curves that are closer to the diagonal dashed line. **b)** The Mean Decrease Gini of all the predictors in the RF model. A higher Gini score signifies higher importance of the variable to the predictive model.

To evaluate whether LoF calls from a single reference genome can serve as a proxy for gene dispensability in the pan-genome, we trained a random forest model to predict dispensability using gene features. Input features included LoF frequency (from Col-0 calls), tissue breadth, epigenomic patterns, and sequence composition, and the dispensability status of each gene was derived from a recent Arabidopsis pan-genome study of 32 genotypes (Kang et al. 2023), where core genes were classified as “indispensable” whereas softcore genes, dispensable genes and private genes were classified as “dispensable” in the random forest model. The model achieved strong predictive performance (AUC-ROC = 0.811, Fig. 1a). Among all features, LoF frequency was by far the most predictive with the highest Mean Decrease Gini score, followed by tissue breadth and H3K36me3. A model categorizing softcore genes as “indispensable” was also tested, as they clearly represent a high degree of functional importance. This model consistently demonstrated strong and reliable predictive performance (AUC-ROC = 0.842, Figure S2). These results indicate that the frequency of LoF variants called against a single reference genome is highly predictive of whether genes are dispensable in the pan-genome, supporting the utility of single-genome LoF calls as a proxy for broader pan-genome classifications.

### LoF-tolerant and LoF-intolerant genes display distinct features in Arabidopsis

**Figure 2:**
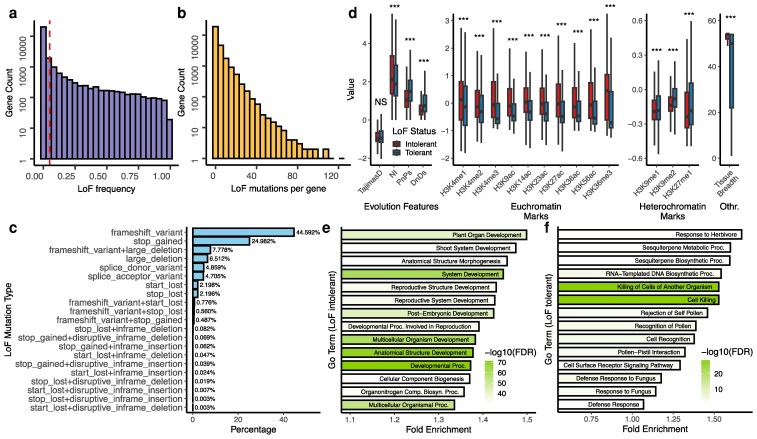
Characteristics of loss-of-function (LoF) mutations in Arabidopsis. **a)** The histogram of LoF frequency of all Arabidopsis genes. Red dash line indicates the 5% allele frequency threshold commonly used in Genome-Wide Association Studies. **b)** The histogram of numbers of independent LoF mutations per gene. **c)** Percentage of each mutation type that causes gene disruption. **d)** Box plots of gene features of LoF-tolerant and LoF-intolerant genes in Arabidopsis. The center line indicates the median; boxes represent the interquartile range (25th–75th percentiles); whiskers extend to 1.5× the interquartile range. Statistical significance was assessed using a two-sided Student's *t*-test. *** indicates *P* < 0.001; NS indicates not significant. **e)** Top 14 Gene Ontology (GO) terms of LoF-intolerant genes in Arabidopsis ranked by Fold Enrichment. **f)** Significant Gene Ontology (GO) terms of LoF-tolerant genes in Arabidopsis ranked by Fold Enrichment.

Most genes have low LoF frequency across all accessions, and 10,999 genes remain functional (LoF frequency = 0) in all 1,113 accessions represented in the LoF allele matrix (Fig. 2a). To better understand the distribution of LoF mutations, we categorized genes by their mutational burden: 10,873 genes (39.7%) carry no predicted LoF mutations in their gene body, 5,278 genes (19.3%) harbor only one, and the remaining 41.0% contain multiple independent LoF mutations (Fig. 2b). Five genes have more than 100 independent LoF alleles accumulating in the natural population, and they are either annotated as hypothetical proteins/pseudogenes or predicted protein-coding genes lacking protein-level evidence. It is likely that genes accumulating many LoF variants are undergoing pseudogenization. When considering the mutations that lead to loss of function, the most prevalent types of LoF variants we detected were frameshift mutations and premature stop codons, which represent 44.59% and 24.98% of all variants, respectively (Fig. 2c), consistent with previous reports (Xu et al. 2019).

Chromatin states, such as histone modifications, are closely linked to gene regulation and function (Berr et al. 2011; Deal and Henikoff 2011; Liu et al. 2018). Because these epigenomic features can reflect the functional importance of genes, we investigated whether gene tolerance to loss of function is associated with specific chromatin marks. Genes were categorized into two groups according to their tolerance to loss of function (tolerant: LoF frequency > 0). We found that genes intolerant to LoF are enriched in H3K4me1, H3K4me2, H3K4me3, H3K9ac, H3K14ac, H3K23ac, H3K27ac, H3K36ac, H3K36me3, and H3K56ac (Fig. 2d). These epigenomic marks are generally associated with active gene transcription (Roth et al. 2001; Bannister and Kouzarides 2011; Deal and Henikoff 2011; Liu et al. 2018; Lee et al. 2020; Jamge et al. 2023). However, the same enrichment was not observed in H3K27me1, H3K9me1, and H3K9me2, which have been previously shown to be linked to gene repression or heterochromatin formation (Noma K et al. 2001; Schotta et al. 2002; Deal and Henikoff 2011; Pan et al. 2018). These findings are similar to those in humans, where loss-of-function intolerant genes are enriched for active epigenomic marks (Boukas et al. 2022). We further observed higher Pn/Ps, higher Dn/Ds ratios, and lower neutrality index (NI) (McDonald and Kreitman 1991) in genes tolerant to LoF, consistent with these genes experiencing less purifying selection and reduced functional constraint compared to genes intolerant to LoF. LoF-intolerant genes are also expressed in a wider range of tissue types (higher tissue breadth), indicating their roles across diverse biological processes and developmental stages. Interestingly, we also found that genes highly expressed in reproductive tissues exhibit significantly lower LoF rates compared to the genome-wide average (Figure S3a). This underscores the critical importance of reproduction-related genes, which appear to be under stronger selective pressure. As expected, genes with prominent mutant phenotypes (Lloyd and Meinke 2012) also exhibit lower LoF frequencies compared to other genes (Figure S3b).

Moreover, we performed functional analysis of LoF-tolerant and LoF-intolerant genes (Table S1) using Gene Ontology (GO) terms. LoF-intolerant genes are enriched with functionally important GO terms that are vital to plants' growth, reproduction, and development (Fig. 2e). This is expected as detrimental LoF mutations in functionally important genes would have been purged by purifying selection. Conversely, the GO terms for LoF-tolerant genes (LoF frequency > 0) are enriched in sesquiterpene metabolic process and defense-related GO terms, including “Killing of Cells of Another Organism” and “Defense Response” (Fig. 2f). Sesquiterpenes are a diverse group of naturally occurring terpenes and play crucial roles in plants, including defense mechanisms, environmental stress responses, communication and signaling, etc (Chizzola 2013). Plants maintain a delicate balance in allocating energy and resources between defense and growth (Giolai and Laine 2024). Previous research found that a mutation in *Replication Factor C Subunit 3* (*RFC3*) enhances resistance to pathogen infection but also leads to developmental defects, including smaller plant size, narrower leaves and petals, and reduced cell proliferation (Xia et al. 2009). In certain environments, it may be advantageous for plants to lose genes associated with defense mechanisms, enabling greater allocation of resources toward overall growth and reproduction. The regulation of biotic and abiotic stress responses is highly interconnected, with extensive signaling cross-talk shaping plant adaptation (Jambunathan et al. 2001; Paparella et al. 2014; Desaint et al. 2024). Recent studies highlight this trade-off: a disease resistance gene in wild pepper was repeatedly lost at high temperatures, indicating that pathogen resistance may entail fitness costs (Poulicard et al. 2024), while species with lower pathogen pressure exhibit pronounced loss of immune receptors during adaptation (Li et al. 2025). Moreover, the convergent loss of plant immunity components across multiple lineages underscores the complex coevolutionary interplay between the immune system and drought tolerance, as these genes are differentially regulated in defense and drought response pathways (Baggs et al. 2020). Interestingly, some cases of gene loss enhance immunity without compromising growth and architecture (Castelló et al. 2010; Wang et al. 2019), making these genes particularly valuable for agricultural applications.

LoF-tolerant genes in Arabidopsis are also associated with “Rejection of Self Pollen”, “Recognition of Pollen”, and “Pollen–Pistil Interaction”. Because Arabidopsis is a dominantly inbreeding plant species, the predominance of inbreeding may reduce the need for or even select against the function of genes that mediate pollen–pistil interactions and self-incompatibility. Notably, the GO terms associated with LoF-tolerant genes show substantial overlap with those of genes affected by copy number variations (CNVs) as reported by Zmienko et al. (2020). These terms are significantly enriched for processes related to interactions with other organisms, defense mechanisms, and stress responses. We also permuted the LoF-tolerant and LoF-intolerant gene lists, and no significant enrichment was observed for either permuted list (Table S1), supporting the robustness of our GO analysis results. Furthermore, LoF mutations are more commonly tolerated in non-single-copy genes, as expected (Xu et al. 2019) (Figure S3c).

We also observed a positive correlation between gene CDS length and the number of accumulated LoF variants (*P* < 2.2 × 10^−16^), which appears to depend on the functional importance of the gene. However, despite this overall trend, some genes accumulate relatively few LoF variants regardless of their length. This may indicate that these genes are under stronger selective pressures, possibly due to their constrained biological functions. (Figure S4).

### Within- and cross-Species prediction of LoF tolerance through epigenomic patterns

**Figure 3:**
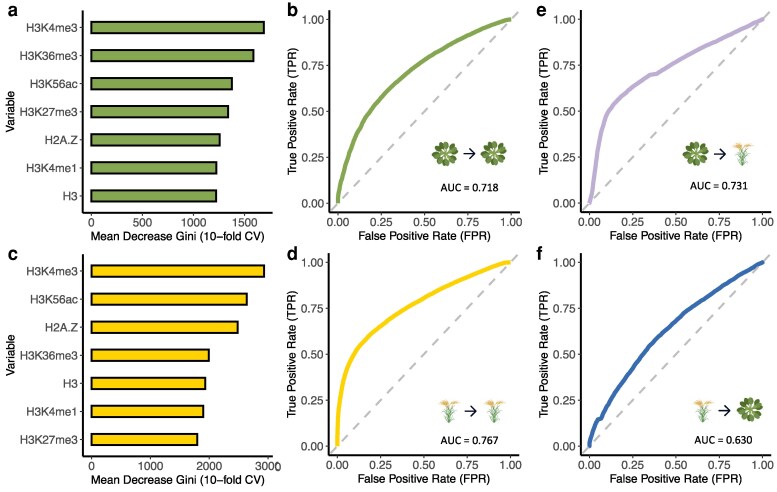
Within- and cross-species prediction of LoF tolerance through epigenomic patterns. **a)** The Mean Decrease Gini of histone marks in predicting Arabidopsis LoF tolerance. **b)** The ROC curve of the random forest model trained on Arabidopsis thaliana histone marks to predict Arabidopsis LoF tolerance. **c)** The Mean Decrease Gini of histone marks in predicting rice LoF tolerance. **d)** The ROC curve of the random forest model trained on rice histone marks to predict rice LoF tolerance. **e)** The ROC curve of the Arabidopsis-trained random forest model tested on rice data. **f)** The ROC curve of the rice-trained random forest model tested on Arabidopsis data. For **b**, **d**, **e** & **f)**, the diagonal dashed line shows the expected performance of a model based on random guessing. Curves closer to the upper left corner of the chart represent a better predictive performance than curves that are closer to the diagonal dashed line.

Histone modifications are well established as regulators of gene activation and repression (Bannister and Kouzarides 2011; Berr et al. 2011; Deal and Henikoff 2011; Liu et al. 2018). More recently, they have also been implicated in DNA repair and mutation probabilities, which can in turn shape the patterns of gene LoF (Monroe et al. 2022; Quiroz et al. 2024). Building on this, we next set out to test if epigenomic information could serve as a broadly applicable predictor of gene LoF tolerance in plants. Using a random forest model trained with *Arabidopsis thaliana* histone marks, we achieved a moderate predictive performance for gene LoF tolerance within the species, with an AUC-ROC of 0.718 and H3K4me3 being the top predictor (Fig. 3a and b). We applied the same approach to rice (*Oryza sativa*) by training a separate random forest model with its corresponding histone marks, achieving a similar result with an AUC-ROC of 0.767 and H3K4me3 being the most important predictor (Fig. 3c and d). These results indicate that epigenomic features can be used to infer LoF tolerance. To further evaluate the generalizability of these models, we tested them across species. When the Arabidopsis-trained model was applied to rice data, it achieved an AUC-ROC of 0.731 (Fig. 3e), comparable to within-species predictions for Arabidopsis. Conversely, the rice-trained model tested on Arabidopsis data yielded a lower AUC-ROC of 0.630 but still outperformed a random expectation (Fig. 3f). This cross-species validation underscores the potential of conserved epigenomic features as reliable predictors of gene LoF tolerance across diverse plant species. Interestingly, the Arabidopsis-trained model predicted LoF tolerance in rice nearly as well as it did within Arabidopsis, whereas the rice-trained model did not transfer well to Arabidopsis, despite performing strongly within rice. One possible explanation is that LoF calls in rice may be less comprehensive or accurate. In our analysis, rice LoF variants were restricted to high-effect annotations from SnpEff, which may underestimate true LoF variation compared to the more extensively curated Arabidopsis dataset. Another contributing factor may be that species-specific relationships between epigenomic features and LoF tolerance are stronger in rice, which would limit the generalizability of rice-trained models to Arabidopsis. We emphasize that the exact explanation remains uncertain, but that these are potential possibilities.

### Collapsing LoF alleles into their functional states mitigates allelic heterogeneity in association testing

**Figure 4:**
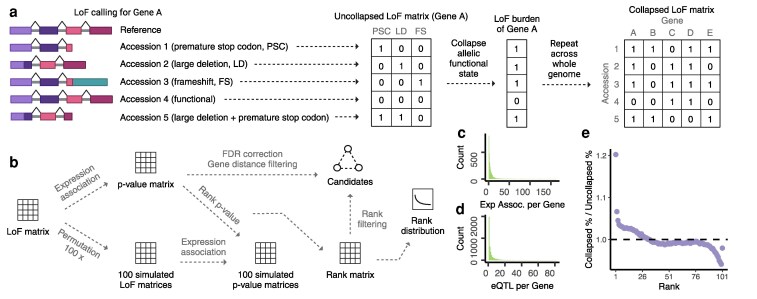
Collapsing LoF alleles into LoF burdens mitigates allelic heterogeneity. **a)** A diagram illustrating LoF calling from a single reference genome and the creation of the LoF matrix. The LoF matrix reflects a binary state of LoF or functional for each accession–gene combination, so independent LoF alleles in the species are collapsed into a single allele state, where 1 indicates LoF, and 0 indicates a functional allele. **b)** An illustration of the LoF-expression association workflow and candidate filtering process. **c)** The histogram of the number of expression associations per LoF gene. **d)** The histogram of the number of eQTLs per expression gene. **e)** The ratio of association percentage at different ranks for collapsed and uncollapsed LoF association testing. More high-ranking associations were identified with the LoF burden approach. Dash line represents null expectation (ratio = 1).

Gene loss of a locus can be caused by multiple independent molecular variants, for example, a frameshift mutation or the introduction of a premature stop codon. This phenomenon is called allelic heterogeneity and can be problematic for the conventional functionally agnostic genome-wide association studies, potentially masking important loci (Monroe et al. 2021). Categorizing variants by their predicted functional effects should theoretically increase our ability to detect causative loci exhibiting allelic heterogeneity. Building on this idea, loss-of-function burden tests have been developed to aggregate the effects of multiple rare or independent LoF variants within a gene into a single burden score. By collapsing these variants at the gene level, this approach enhances statistical power to detect associations between gene disruption and phenotypic variation, even when individual variants are too rare to show significant effects on their own (Povysil et al. 2019). Based on the prediction of variant effects on gene function, we created uncollapsed and collapsed LoF matrices, respectively (***Materials and methods***, Fig. 4a). Gene expression as a class of quantitative traits can be highly heritable and have complex genetic architecture (Kliebenstein 2009). In this study, we utilized gene expression as a suite of quantitative traits to evaluate the performance of LoF burden tests for gene discovery in association studies in plants, testing for the effects of candidate loci on the expression of thousands of genes at a time. Using the collapsed LoF matrix and transcriptome data from the 1001 Genomes collection of *Arabidopsis thaliana* (Kawakatsu et al. 2016), we performed genome-wide LoF burden-expression tests, which yield 14,175 significant associations after stringent filtering processes (***Materials and methods***, Fig. 4b). The loss of function of one gene is associated with the expression of up to 175 other genes, although the majority of LoF genes are associated with the expression of fewer than 50 genes (Fig. 4c). Similarly, individual expressing genes are associated with up to 80 expression quantitative trait loci (eQTLs), with most genes linked to fewer than 20 eQTLs (Fig. 4d). Statistically, the power to detect associations increases with the allele frequency of a variant, which is why common genetic variants have been extensively studied, while rare variants remain a compelling frontier in GWAS (Lee et al. 2014; Auer and Lettre 2015). Interestingly, we observed a higher number of significant LoF-expression associations for genes with either very low or very high LoF frequencies (Figure S5). A possible explanation for this pattern is that genes with low LoF frequencies are likely to be functionally pleiotropic, and their loss is more likely to affect the expression of numerous other genes. Alternatively, this result could reflect a bias in the detection of non-normally distributed phenotypes with rare variants (Beasley et al. 2009; McCaw et al. 2020), which we therefore aimed to address.

To further reduce false positives and eliminate the bias of allele frequency, the *P*-value of each association test was ranked among the first 100 simulations, and the 101st simulation was also ranked among the same 100 simulations to generate a null distribution of the rank matrix (Fig. 4b). An enrichment of high ranks (eg rank 1 to 10) was observed in the LoF-expression associations while the null scenario is evenly distributed (Figure S6), suggesting more significant associations between gene LoF and gene expression than expected by chance. To test if collapsing LoF alleles mitigates allelic heterogeneity and, as a result, increases the power of association testing, we performed the same approach with the uncollapsed LoF allele matrix. Overall, a reduction in high-rank and an increase in low-rank LoF-expression associations were observed with the uncollapsed LoF allele associations. Specifically, there was a 17.2% decrease in the percentage of rank 1 associations (Fig. 4e, Figure S6). This aligns with the expectation that LoF burden approaches, which contrast alleles based on their functional state rather than individual variants or linked SNPs, have improved power by overcoming allelic heterogeneity. Intriguingly, we observed a slight enrichment of rank 101 associations in both collapsed and uncollapsed tests. This pattern is likely attributable to correction for genetic relatedness in a GWAS framework, caused by extreme covariance between allele states and the kinship matrix. To further investigate, we performed associations without accounting for kinship, using a simple linear regression model. While this approach resulted in no enrichment at low ranks (eg rank 90 to 101) and a much higher number of rank 1 associations, as expected for true positives, many still are likely false positives driven by spurious correlations due to genetic relatedness (Figure S6).

### LoF-expression associations recapitulate the canonical flowering regulatory network

**Figure 5:**
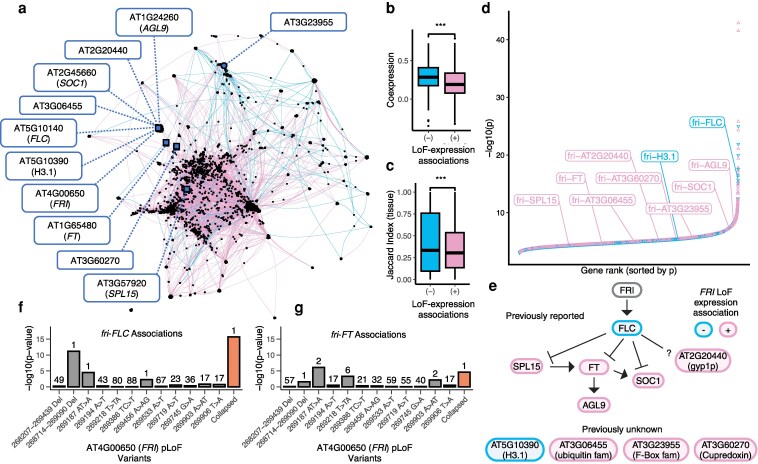
LoF burden-expression association recapitulates the canonical flowering regulatory network. **a)** The gene network constructed from LoF burden-expression association results. Nodes in the network represent genes, categorized as either LoF genes (circles) or Exp genes (squares). Genes serving as both LoF and Exp genes are depicted as circles. The edges denote directed associations from LoF genes to Exp genes, with edge colors indicating the direction of the association: pink for positive associations and blue for negative associations. Flowering time network genes identified by LoF burden-expression association are highlighted with navy blue nodes and labels. **b)** Coexpression coefficients of negative and positive LoF-expression association gene pairs. c) Jaccard Index of tissue-specific expression profiles of negative and positive LoF-expression association gene pairs. A higher Jaccard Index means greater similarity between the tissue-specific expression profiles of two genes. For **b)** & **c)**: The center line indicates the median; boxes represent the interquartile range (25th–75th percentiles); whiskers extend to 1.5× the interquartile range; points denote outliers. Statistical significance was assessed using a two-sided Student's *t*-test. *** indicates *P* < 0.001. **d)** LoF-expression associations ranked by -log10(*P*). Positive (pink) and negative (blue) associations are distinguished, with *FRI* associations labeled. **e)** The flowering regulatory network described in previous studies and signified by LoF burden-expression association. Arrows indicate gene activation, and blunted lines indicate repression. Pink: positive expression associations with *FRI* LoF; Blue: negative expression associations with *FRI* LoF. **f** & **g)** The uncollapsed and collapsed association results for *fri*-*FLC* and *fri*-*FT* associations, respectively. Bars indicate uncollapsed (gray) and collapsed (orange) association significance, with x-axis labels showing variant positions or deletions. Numbers above bars indicate the rank of LoF-expression associations across 100 simulations.

In the global analysis of LoF-expression associations, we identified 11,445 significant positive (eg LoF in gene A is associated with increased expression in gene B) and 2,730 negative associations for a ratio of positive/negative of 4.2 (Table S2). These associations can be visualized as a gene network that captures the significance and directionality of each association (Fig. 5a). The observed enrichment of positive LoF-expression associations could result from repressor genes being more tolerant to loss of function, or from a detection bias in RNA-seq favoring highly expressed genes with greater statistical power. To assess whether the unbalanced ratio of positive to negative associations has biological significance or is merely a statistical artifact, we applied the same filtering process to 50 simulated datasets generated by permuting LoF and functional alleles. Surprisingly, the simulations produced even higher positive-to-negative association ratios (Figure S7a). This indicates that the inflation of positive associations could be largely driven by artifacts in the association testing. However, the number of biologically meaningful positive LoF-expression associations we observed is actually lower than expected by chance, suggesting that the real data may be less influenced by statistical noise than the permuted datasets and therefore more reflective of true underlying biological relationships. To explore whether the non-normal distribution of expression data contributes to this bias, we applied a rank-based inverse normal transformation (INT) (McCaw et al. 2020). However, INT did not fully normalize the expression data, as 8,589 genes (37.5% of all expressed genes) remained non-normally distributed due to an abundance of zero-value observations (Shapiro-Wilk normality test, *P* < 0.05). After excluding these genes, the positive-to-negative association ratio dropped to 0.63, while the 50 simulations averaged 1.06 (One-Sample *t*-test, *P* < 2.2 × 10^−16^, Figure S7b). These findings suggest that the observed bias toward positive associations is driven by enrichment of type I errors for positive associations due to the skewed distribution of the expression phenotype and unequal group sizes in the binary predictor, which should be carefully considered in future eQTL studies, particularly when analyzing associations with rare alleles. Additionally, the higher prevalence of negative LoF-expression associations suggests that many genes may function to promote the expression of others, consistent with widespread positive regulatory interactions in the transcriptome. An additional source of potential bias is that LoF variant detection and RNA-seq expression estimates may both be affected by genetic distance from the Col-0 reference, such that read alignment in divergent accessions could artificially increase the apparent number of negative LoF-expression associations.

Given the concerns about the imbalance between positive and negative associations, we next asked whether the directionality of these associations aligns with known gene functional relationships inferred from independent datasets. Subsequently, we constructed gene coexpression networks using Spearman correlation based on gene expression data. The global patterns revealed that gene pairs with negative LoF-expression Beta coefficients exhibit higher coexpression coefficients compared to those with positive Beta coefficients (Fig. 5b). To explore the spatial linkages of these significant gene pairs, we calculated the Jaccard similarity index of their tissue-specific expression profiles, finding that gene pairs with negative Beta coefficients also tend to be expressed in similar tissues (Fig. 5c). A gene pair with a negative LoF-expression Beta coefficient is positively co-regulated—perhaps part of the same pathway or functional module, where the activity of one gene supports or enhances the activity of the other (Eisen et al. 1998). It is natural that they are also expressed in similar tissues as genes involved in the same biological processes often exhibit tissue-specific coexpression because their function is required in specific cell types or environmental conditions (Marbach et al. 2016; Sonawane et al. 2017). On the other hand, genes in antagonistic relationships are less likely to be directly coexpressed because their regulation is inverse rather than synchronous. Consequently, lower coexpression coefficients are expected for gene pairs with positive Beta coefficients. These findings underscore a strong functional and spatial connection between significantly associated LoF-expression gene pairs, suggesting their potential involvement in coordinated biological processes.

Due to the poor annotation of many less-studied non-essential genes, interpreting many of these associations remains challenging. Nevertheless, the LoF burden-expression tests effectively captured associations between *FRIGIDA* (*FRI*), a major determinant of natural variation in Arabidopsis flowering time, and its downstream targets in the well-studied flowering time pathway. These associations exhibit remarkable statistical significance, with directionalities that align with findings from previous empirical studies (Fig. 5d and e). Specifically, the LoF of *FRI* is negatively correlated with the expression of *FLOWERING LOCUS C* (*FLC*), consistent with earlier reports that *FRI* activates *FLC* transcription (Clarke and Dean 1994; Gazzani et al. 2003; Choi et al. 2011). Additionally, positive associations were observed between the LoF of *FRI* and the expression of *FLOWERING LOCUS T* (*FT*) and *SUPPRESSOR OF OVEREXPRESSION OF CONSTANS1* (*SOC1*), which is consistent with findings that *FT* facilitates the activation of *SOC1* (Yoo et al. 2005) and that *FLC* binds and represses *FT* and *SOC1* (Deng et al. 2011).

LoF burden-expression tests also identified a positive association between *FRI* LoF and *AGAMOUS-LIKE 9* (*AGL9*) expression, a gene downstream of *FT* that promotes flowering (Mandel and Yanofsky 1998; Yu and Goh 2000; Hsu et al. 2003; Gao et al. 2021). Similarly, a positive association was noted with *SQUAMOSA PROMOTER BINDING PROTEIN-LIKE 15* (*SPL15*), known to promote *FT* (Kim et al. 2012), mediate floral transition (Xu et al. 2016), and which is repressed by *FLC* (Deng et al. 2011). Another notable association is with the expression of AT2G20440, a Ypt/Rab-GAP domain protein whose knockout results in early flowering (Chien et al. 2023), though the molecular mechanism and its interactions with other flowering genes warrant further investigation.

Furthermore, our results uncovered associations involving *FRI*, including genes such as AT5G10390 (HISTONE 3.1), AT3G06455 (ubiquitin family protein), AT3G23955 (F-box family protein), and AT3G60270 (Cupredoxin superfamily protein). Notably, LoF burden-expression tests not only detected direct targets of the LoF gene but also downstream pathway components, exemplified by capturing associations between *FRI* and *FT*, with *FLC* as an intermediary (Fig. 5d). These findings highlight the power of LoF burden-expression tests to dissect complex regulatory networks, offering insights into gene interactions and uncovering both known and previously uncharacterized biological connections.

Because the *fri*-*FLC* relationship serves as a positive control whose causality is extensively supported experimentally, it served as a test case for examining the consequence of allelic heterogeneity in gene discovery. We therefore compared the uncollapsed LoF-expression association results to the collapsed LoF burden approach, focusing on associations involving *FRI*. When associating with *FLC* expression, collapsing LoF alleles resulted in a notably reduced *P*-value, underscoring the increased sensitivity of the collapsed approach. Interestingly, the strongest signal in the uncollapsed association testing was driven by a large structural deletion (Fig. 5f). This finding is particularly striking, as structural variations have often been underexplored in traditional GWAS analyses. In the case of the *FT* association, the collapsed LoF burden test produced a slightly less significant *P*-value compared to the most significant uncollapsed variant. However, the LoF burden approach achieved a higher rank across simulations (Fig. 5g), suggesting greater robustness in identifying biologically relevant associations. Overall, these observations highlight that collapsing alleles with shared functional consequences may reduce noise and better capture the cumulative effects of LoF variants on trait variation. This further supports the utility of collapsing LoF alleles in LoF burden tests as a strategy to uncover subtle but meaningful genetic associations, particularly in traits influenced by complex genetic architectures.

### LoF burden tests with flowering time capture *FRIGIDA* as a key regulator

**Figure 6:**
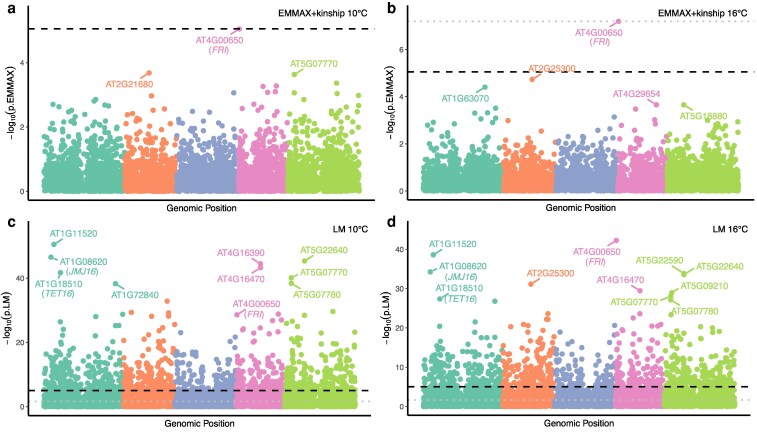
LoF burden tests with flowering time capture *FRIGIDA* as a key regulator. **a** & **b)** Manhattan plots showing genome-wide LoF burden tests using EMMAX with flowering time at 10 °C and 16 °C, respectively. Each point represents the LoF burden test for a specific gene, with colors indicating the chromosome on which the gene is located. The genomic position of each gene is determined as the midpoint between its start and end positions, based on the Col-0 gene annotation. The dashed black line indicates the significance threshold determined by Bonferroni correction, with *FRIGIDA* (AT4G00650) being the only gene surpassing this threshold. Dotted grey lines represent the largest significant FDR-adjusted *P*-value. **c** & **d)** Manhattan plots showing genome-wide LoF burden tests using a linear model approach with flowering time at 10 °C and 16 °C, respectively. A substantial number of associations exceeded both Bonferroni and FDR thresholds.

Inspired by the identification of the flowering time network through LoF burden tests with gene expression data, we sought to investigate whether association tests with phenotypic traits, such as flowering time, could yield similar insights. To explore this, we conducted genome-wide LoF burden tests for flowering time at 10 °C and 16 °C (1001 Genomes Consortium 2016) using EMMAX. As anticipated, *FRI* LoF emerged as a highly significant gene for flowering time at both temperatures, with results nearing or surpassing a stringent Bonferroni-corrected *P*-value threshold. However, no other significant associations were identified under these conditions (Fig. 6a and b). In addition to modeling kinship as a random effect, incorporating the top five principal genetic components as fixed effects produced nearly identical results (Figure S8 and S9), suggesting that the kinship matrix already captures most of the underlying population structure.

In GWAS, correction for kinship or population structure can obfuscate true causal relationships. This challenge is especially pronounced for flowering time, since the trait itself contributes to non-random mating and thus generates structured populations. In such cases, corrections for kinship or population structure may disproportionately remove true associations, making it particularly difficult to disentangle confounding from genuine biology. To evaluate whether relaxing structure control uncovers additional meaningful signals, we tested association models with different levels of control for kinship and population structure (***Materials and methods***). Specifically, we performed a simplified version of the LoF burden tests using linear models, with or without the top five PCs as covariates. Under this less stringent approach, a considerable number of associations surpassed the Bonferroni and FDR threshold, in addition to *FRI* (Fig. 6c and d, Figure S8). Distinguishing whether these signals represent false positives due to uncorrected population stratification or whether they are genuine biological associations is challenging, as the Quantile–Quantile plot remained severely inflated even after incorporating the top five PCs (Figure S9). A notable case in this regard is AT1G11520, which appeared among the most significant hits at both 10 °C and 16 °C in the linear models. Previous research by Liu et al. (2021) used a deletion-based genome-wide association approach and found that accessions carrying a 182-bp deletion in AT1G11520 exhibited delayed flowering. Interestingly, all accessions from northern Sweden contained this deletion, highlighting a strong population structure. This high degree of population stratification likely explains why this signal was lost when structure was controlled for in our analysis. However, we acknowledge that D.-X. Liu et al. (2021) analysed the same Arabidopsis 1001 Genomes dataset, and this association has not been confirmed in independent datasets or through experiments. Other highly significant associations revealed here also yield compelling candidates (Table S3). For example, multiple MADS-box genes show associations with flowering time at 10 °C, including AT2G41445 (*P* = 6.43 × 10^−30^), AT2G41440 (*P* = 6.71 × 10^−29^), AT4G11880 (*P* = 9.69 × 10^−12^), and AT2G41470 (*P* = 1.84 × 10^−9^), etc. Members of the MADS-box gene family are well known for their roles in the transition to flowering (Parenicová et al. 2003). In particular, AT4G11880 (*AGAMOUS-LIKE 14*, *AGL14*) has previously been shown to promote flowering (*Pérez-Ruiz et al. 2015*; Chen et al. 2022), aligning with our finding that loss-of-function of *AGL14* is associated with increased flowering time, despite its low LoF allele frequency (LoF frequency = 0.036).

Similarly, loss of function in AT1G08620 (*JUMONJI DOMAIN-CONTAINING PROTEIN 16*, *JMJ16*) is significantly and positively associated with flowering time at both 10 °C and 16 °C (Fig. 6c and d, Figure S8), suggesting a role for *JMJ16* in promoting flowering. The Arabidopsis genome encodes six homologs of Lysine Demethylase 5 (KDM5): *JMJ14*, *JMJ15*, *JMJ16*, *JMJ17*, *JMJ18*, and *JMJ19* (Lu et al. 2008). While previous studies reported no discernible phenotype for *JMJ16* mutants in the Col-0 background (Yang et al. 2010), other members of this family—such as JMJ14, which represses the floral transition (Yang et al. 2010), and *JMJ15* and *JMJ18*, which promote flowering (Yang et al. 2012a, 2012b)—support the plausibility of a regulatory role for *JMJ16* as well. It is possible that the lack of a phenotype previously observed in the Col-0 background reflects epistatic interactions with other loci, such as the inactive *FRIGIDA* allele in Col-0 (Johanson et al. 2000; Risk et al. 2010), which may mask flowering-related effects of *JMJ16* loss of function.

Another noteworthy candidate is AT1G18510 (*TETRASPANIN 16*, *TET16*), which also shows a significant association with flowering time at both 10 °C and 16 °C (Fig. 6c and d, Figure S8). While the role of tetraspanins in plant development remains poorly understood, prior studies suggest they may contribute to reproductive processes in flowering plants (Wang et al. 2012, 2015; Boavida et al. 2013). Previous expression and regulatory analyses have implicated *TET3* in the flowering response under low temperatures, including interactions with key flowering time regulators such as *PSEUDO-RESPONSE REGULATOR 5* (*PRR5*), *AGAMOUS-LIKE 15* (*AGL15*), and PHYTOCHROME INTERACTING FACTOR 4 (PIF4) (Wang et al. 2015). The identification of *TET16* in the flowering time association suggests it may have a role in flowering regulation. We should also emphasize that although we cannot fully exclude the possibility of spurious associations due to limited control for kinship or population structure in these cases, the strength of these associations suggests they may reflect true biological effects and thus merit further investigation.

### Current limitations and potential improvements

Our LoF calling strategy, while effective, is not without its limitations. Using a single reference genome simplifies analyses but inherently limits our ability to capture variation across diverse genomes. Genes absent in the reference genome or structural rearrangements that deviate from it cannot be accurately assessed. For example, tools like SnpEff rely on genome annotations that, while powerful, cannot perfectly predict the functional impact of all variants. Furthermore, our approach considers genetic variants individually. In rare cases, LoF effects may be rescued by compensatory mutations, such as a frameshift mutation restored by a subsequent deletion, which can lead to functional outcomes.

However, as a famous saying goes, “All models are wrong, but some are useful” (Box 1976), our genome-wide LoF burden tests successfully captured the textbook flowering regulatory networks centered around *FRI*, validating its usefulness. Notably, a naturally occurring 16-base-pair deletion is found in the loss-of-function *FRI* allele in Col-0 (Johanson et al. 2000; Risk et al. 2010). This demonstrates that even with imperfect reference-based predictions, functional outcomes of genetic variants can still be inferred. The *FRI* associations, while not the most statistically significant among all LoF-expression association results (Fig. 5d), exemplify the power of LoF burden tests to uncover biologically meaningful signals. This suggests that our dataset of LoF-expression associations represents a unique resource that may contain other true biological interactions warranting further exploration (Table S2).

To address some of the challenges mentioned above, several improvements can enhance LoF prediction in future studies. To begin with, transitioning from single-reference genomes to pan-genomes would enable better representation of genetic diversity across populations, capturing dispensable genes that are absent in the reference. Moreover, integrating tools like AlphaFold could help predict how specific genetic variants impact protein function, providing a more precise definition of LoF.

An intrinsic limitation of our methodology is the statistical challenge posed by low allele frequency. In standard GWAS, detecting significant signals is generally easier when the minor allele frequency (MAF) of a variant is higher. Variants with a MAF below 5% are often excluded from analysis to maintain statistical power and minimize false positives. To ensure the robustness of our candidate selection, we adopted this approach in our study. However, this filtering led to the removal of a large proportion of genes (Fig. 2a), particularly those essential for plant survival. For instance, while we successfully identified *FRI* as a key regulator of flowering time through LoF burden association testing, other well-known regulators such as *FLC* and *FT* were not captured because they did not meet the 5% MAF threshold. That is, the function of genes with few or no natural LoF cannot be studied with this kind of analysis. This limitation is particularly relevant for LoF-intolerant genes, which are often involved in critical biological processes, exhibit low LoF frequencies, and are consequently underrepresented in LoF association testing. These findings align with the recent report by Spence et al. (2026), which highlights that LoF burden tests often fail to prioritize genes based on their importance to a trait. This is because the most trait-critical genes are typically highly constrained and have the lowest LoF variant frequencies. Additionally, Spence et al. noted that gene length significantly influences the power of LoF burden tests, consistent with our observation that coding sequence length is positively correlated with the number of accumulated LoF variants in Arabidopsis. On the other hand, this underscores the importance of accurate LoF variant calling and the aggregation of all LoF variants, as these steps can substantially increase the LoF frequencies of genes of interest, improving their representation in association studies.

## Conclusion and future directions

In this study, we explored the characteristics and functional significance of genes tolerant of accumulating putative loss-of-function alleles. Although these genes are not essential for plant survival, they often play specialized roles in processes such as defense and environmental adaptation. Their functions, however, remain underexplored due to functional redundancy and their non-essential nature. To address this, we constructed a comprehensive LoF allele matrix using available whole-genome sequencing data, detailing the functional status of every gene across all accessions represented in the 1001 Genomes Project (Table S4), in the hope that this LoF matrix will serve as a valuable resource for advancing Arabidopsis genetic studies. Building upon a previous insightful study (Xu et al. 2019) on Arabidopsis LoF variation, our work further incorporates structural variants—an important but sometimes underrepresented source of gene disruption—into LoF calling, and consolidates all LoF variants into gene-level LoF burdens to overcome allelic heterogeneity. We then applied a function-based burden test approach, leveraging gene expression and flowering time as phenotypes to evaluate its effectiveness. Our LoF burden tests successfully identified associations between gene pairs, including those within well-characterized flowering time networks and previously uncharacterized genes, offering insights into both previously undescribed and well-established functional connections.

Our framework for LoF burden tests was developed in Arabidopsis, but future applications will need to account for the added genetic complexity of plant species with high heterozygosity or polyploidy. In such genomes, distinguishing true LoF variants from technical artifacts can be complicated by multiple homeologous or paralogous gene copies, making accurate read mapping and variant calling more challenging. Moreover, functional redundancy in large gene families, where LoF alleles may segregate in a dosage-dependent manner, may mask the phenotypic consequences of individual gene loss, reducing the power to detect meaningful associations. Extending this approach to crops with highly duplicated genomes will therefore require strategies that integrate gene copy-number variation, dosage effects, and subgenome-specific regulation. For applied research, understanding how LoF variants affect agriculturally relevant traits could have significant implications for plant breeding by accelerating the discovery of targets for CRISPR-mediated knockouts. Could we manipulate dispensable genes or create synthetic LoF alleles to improve stress resilience, yield, or nutrient use efficiency? These questions not only provide exciting avenues for future exploration but also underline the importance of studying LoF variants within an integrated framework that connects evolutionary biology, functional genomics, and agricultural applications.

## Materials and methods

### Arabidopsis LoF calling and LoF matrix

Loss-of-function variants were called from publicly available Whole-Genome Sequencing data, including SNP variants and small indel calls for 1,135 Arabidopsis accessions from the 1001 Genomes Project (1001 Genomes Consortium 2016) and structural variants and indel calls for 1,301 accessions (Göktay et al. 2021). The VCF from the 1001 Genomes Project annotated using SnpEff was downloaded from the 1001 Genomes Data Center (https://1001genomes.org/data/GMI-MPI/releases/v3.1/1001genomes_snpeff_v3.1/). The program snpEff (Cingolani et al. 2012) is widely used to annotate mutation effects from genotype variants. Variants annotated as “High” impact are considered as putative LoF variants. Those include “stop_lost”, “stop_gained”, “start_lost”, “frameshift”, “splice_donor_variants”, and “splice_acceptor_variants”. For structural variants, we mainly focus on deletions as they constitute the majority of the data. A deletion variant is considered a LoF mutation if it either causes a frameshift by disrupting the reading frame (ie, the deletion overlaps the coding DNA sequence in a length not divisible by three) or removes more than 10% of the coding sequence of the affected transcript. It is also assumed that the protein product could still be functional with a change in the first and last 5% of the coding sequence, as an alternative start/stop codon may rescue the disrupted transcript, and thus variants that only affect the first and last 5% of the coding sequence were therefore systematically removed (MacArthur et al. 2012). Lastly, to ensure true knockout of a gene and reduce false positives, only variants that disrupt all gene models for the gene of interest are kept. With the prediction of LoF alleles, a LoF allele matrix is generated to reflect the relationship between accessions and LoF alleles. Independent LoF alleles of the same gene were then collapsed into a single allele state. The collapsed LoF allele matrix represents a binary state of LoF or functional for each accession–gene combination, where LoF alleles are shown as “1” and functional alleles are shown as “0” in the LoF allele matrix (Table S4).

### Arabidopsis expression matrix

An expression matrix was generated based on the 1001 Arabidopsis transcriptomes (Kawakatsu et al. 2016) after correction for batch effect. It reflects the log_2_-transformed transcript level for every accession–gene combination. The NaN values after log_2_ transformation were replaced with the lowest transcript value among all accessions for each gene.

To test if the non-normality of expression data changes the scale of LoF-expression associations, we applied a rank-based inverse normal transformation (INT) to normalize non-normally distributed expression data. Non-missing values were ranked (with ties assigned the average rank) and transformed into a standard normal distribution using the inverse cumulative distribution function of the normal distribution. The transformed values retained the original rank order.

### Gene ontology analysis

GO enrichment of LoF-tolerant and LoF-intolerant genes was performed using ShinyGO 0.81 (http://bioinformatics.sdstate.edu/go/) (Ge et al. 2020). An FDR-adjusted *P*-value cutoff of 0.05 was adopted to identify significantly enriched GO terms.

### Arabidopsis gene features and epigenomic data

We compiled and generated data on genome-wide sequence and epigenomic features of Arabidopsis to construct a high-resolution predictive model of pan-genome gene dispensability. Polymorphism was previously calculated from the 1001 genomes project (1001 Genomes Consortium 2016). Tissue breadth data were obtained from Mergner et al. (2020). Additionally, we retrieved 62 BigWig-formatted datasets from the Plant Chromatin State Database (Liu et al. 2018), detailing the distribution of histone modifications, including H3K4me2, H3K4me1, H3K4me3, H3K27ac, H3K14ac, H3K27me1, H3K36ac, H3K36me3, H3K56ac, H3K9ac, H3K9me1, H3K9me2, and H3K23ac. For each modification, signal intensities were normalized (0 to 1) and averaged across each genomic region for downstream analyses.

### Rice LoF-tolerant genes and epigenomic data

To obtain a list of LoF-tolerant genes in rice, a set of 29 million biallelic SNPs with SnpEff annotation was downloaded from the International Rice Research Institute (IRRI) Rice SNP-Seek Database (https://s3.amazonaws.com/3kricegenome/snpseek-dl/NB_bialSNP_pseudo_canonical_ALL.vcf.gz). This dataset consists of 3,024 genotypes and was mapped to the *O. sativa* cv. Nipponbare IRGSP 1.0 reference genome (Wang et al. 2018). Any genes with variants annotated with “High” impact were extracted. To build predictive models of LoF tolerance, the Arabidopsis and rice epigenomic data were obtained from Lu et al. (2019).

### Random forest predictions

Random Forest Predictions in this study were performed using the R package “randomForest”. Pan-genome dispensability and gene LoF tolerance were converted to binary factors. For pan-genome dispensability prediction, a dataset of 25,611 genes with 19 gene features was used. For LoF tolerance prediction through histone marks, 27,206 Arabidopsis genes and 55,986 rice genes with corresponding epigenomic data were included. We applied 10-fold cross-validation with balanced training sets, randomly downsampling the majority class to match the minority class in each fold. The predictors (gene features or histone marks) were fitted into the model with 500 decision trees (ntree = 500). Model performance was assessed using receiver operating characteristic (ROC) curves and the area under the curve (AUC), with feature importance quantified by the Mean Decrease in Gini. All genes were held out exactly once for validation, and predictions and variable importance were aggregated across folds to obtain robust performance metrics. For cross-species predictions, all available data were used for model building after balancing.

### Gene coexpression network and coefficient

To measure gene co-expression, we used previously published data from 874 transcriptomes of natural Arabidopsis genotypes, capturing stochastic interindividual variation. We calculated all-by-all expression correlations using Spearman correlation coefficients, employing the mr2mods program (Kawakatsu et al. 2016; Wisecaver et al. 2017). This data was previously published by Katz et al. (2022).

### Genome-wide LoF burden tests and permutations

Genome-wide association testing through LoF burdens was performed using the EMMAX algorithm (Kang et al. 2010) with the function “cGWAS.emmax” in the R package “cpgen”. The collapsed or uncollapsed binary LoF allele matrix was input as the marker matrix, and the expression level of each gene from the expression matrix was input as a phenotype for each association testing. The kinship matrix derived from SNP data (https://1001genomes.org/data/GMI-MPI/releases/v3.1/SNP_matrix_imputed_hdf5/) was used in the model to control for genetic relatedness.

To mitigate the bias of allele frequency in association studies, we performed 101 permutations for the collapsed and uncollapsed LoF allele matrices, respectively. The order of LoF or functional alleles (“1” or “0” in LoF allele matrix) was shuffled for each gene (or allele for the uncollapsed matrix), but the same allele frequency was maintained. The 101 simulated LoF matrices were input into the LoF association testing pipeline as described above. The *P*-values from the LoF-expression associations were then compared to and ranked among the simulated values from 100 permutations. A rank matrix was obtained, showing the rank of the *P*-value or the absolute value of the beta coefficient among 100 simulations for each LoF-expression association. The 101st simulated *P*-value matrix was also ranked among the 100 simulations to create null rank matrices.

Besides expression association, we also obtained flowering time phenotype at both 10 °C and 16 °C from the 1001 genome project (1001 Genomes Consortium 2016). Association tests with flowering time were modeled with different levels of confounding effect control. Specifically, four commonly used approaches were tested:

1. Naïve linear model (GLM): y=xβ+ε
2. Linear model with top 5 principal components (PCs): y=xβ+Cγ+ε
3. Linear mixed model with kinship using EMMAX: y=xβ+u+ε
4. Linear mixed model with top 5 PCs and kinship using EMMAX: y=xβ+Cγ+u+ε

In these equations, *y* denotes the phenotype of interest, xβ represents the effect of the alleles being tested, and ε is the residual error term. In models that include principal components, Cγ captures the contribution of the top 5 PCs, which account for broad-scale population structure. In models that include kinship, *u* is a random effect with distribution $u∼(0,\sigma_{g^{2}}K)$ , where *K* is the kinship matrix used to model relatedness among individuals. The genetic principal components were calculated from the 1001 genomes VCF using Plink.

### Candidate filtering

To minimize false positives, association hits underwent a series of stringent filtering steps. First, the *P*-values of LoF-expression associations were corrected for multiple comparisons using the false discovery rate (FDR) method implemented in the “p.adjust” function. Associations with an FDR-adjusted *P*-value greater than 0.05 were excluded. Second, to eliminate potential linkage effects, gene pairs located within 1,000 kb of each other were removed. Genes annotated as “hypothetical” were also removed. Finally, only candidates with the top-ranked (rank 1) *P*-value among 100 simulations were retained, ensuring only the most robust associations were considered for downstream analysis. While other high-ranking associations were not included in subsequent analysis, they may still represent biologically meaningful signals.

### Gene network

We constructed and visualized a gene interaction network based on LoF burden-expression associations using the R package “igraph”. Genes are represented as nodes, with edges indicating the relationships between LoF genes and their associated expression genes. The strength of these associations is weighted by statistical significance, while directionality is distinguished based on whether the association is activating or repressing.

## Supplementary Material

koag087_Supplementary_Data

## Data Availability

The data underlying this article are available in the article and in its online supplementary material. Code for this project is maintained at: https://github.com/KehanZhao/ArabidopsisLoF.

## Dive Curated Terms

The following phenotypic, genotypic, and functional terms are of significance to the work described in this paper:

AGL14 Gramene: AT4G11880
AGL14 Araport: AT4G11880

TET16 Gramene: AT1G18510
TET16 Araport: AT1G18510

AGL15 Gramene: AT5G13790
AGL15 Araport: AT5G13790

RFC3 Gramene: AT3G17170
RFC3 Araport: AT3G17170

SPL15 Gramene: AT3G57920
SPL15 Araport: AT3G57920

TET3 Gramene: AT3G45600
TET3 Araport: AT3G45600

FLC Gramene: AT5G10140
FLC Araport: AT5G10140

PIF4 Gramene: AT2G43010
PIF4 Araport: AT2G43010

## References

[koag087-B1] 1001 Genomes Consortium . 2016. 1,135 genomes reveal the global pattern of polymorphism in Arabidopsis thaliana. Cell. 166:481–491. 10.1016/j.cell.2016.05.063. 27293186 PMC4949382

[koag087-B2] Albalat R, Cañestro C. 2016. Evolution by gene loss. Nat Rev Genet. 17:379–391. 10.1038/nrg.2016.39. 27087500

[koag087-B3] Auer PL, Lettre G. 2015. Rare variant association studies: considerations, challenges and opportunities. Genome Med. 7:16. 10.1186/s13073-015-0138-2. 25709717 PMC4337325

[koag087-B4] Backman JD et al 2021. Exome sequencing and analysis of 454,787 UK Biobank participants. Nature. 599:628–634. 10.1038/s41586-021-04103-z. 34662886 PMC8596853

[koag087-B5] Baggs EL et al 2020. Convergent loss of an EDS1/PAD4 signaling pathway in several plant lineages reveals coevolved components of plant immunity and drought response. Plant Cell. 32:2158–2177. 10.1105/tpc.19.00903. 32409319 PMC7346574

[koag087-B6] Bannister AJ, Kouzarides T. 2011. Regulation of chromatin by histone modifications. Cell Res. 21:381–395. 10.1038/cr.2011.22. 21321607 PMC3193420

[koag087-B7] Beasley TM, Erickson S, Allison DB. 2009. Rank-based inverse normal transformations are increasingly used, but are they merited? Behav Genet. 39:580–595. 10.1007/s10519-009-9281-0. 19526352 PMC2921808

[koag087-B8] Beaumont RN, Hawkes G, Gunning AC, Wright CF. 2024. Clustering of predicted loss-of-function variants in genes linked with monogenic disease can explain incomplete penetrance. Genome Med. 16:64. 10.1186/s13073-024-01333-4. 38671509 PMC11046769

[koag087-B9] Bergelson J, Roux F. 2010. Towards identifying genes underlying ecologically relevant traits in Arabidopsis thaliana. Nat Rev Genet. 11:867–879. 10.1038/nrg2896. 21085205

[koag087-B10] Berr A, Shafiq S, Shen W-H. 2011. Histone modifications in transcriptional activation during plant development. Biochim Biophys Acta. 1809:567–576. 10.1016/j.bbagrm.2011.07.001. 21777708

[koag087-B11] Boavida LC, Qin P, Broz M, Becker JD, McCormick S. 2013. Arabidopsis tetraspanins are confined to discrete expression domains and cell types in reproductive tissues and form homo- and heterodimers when expressed in yeast. Plant Physiol. 163:696–712. 10.1104/pp.113.216598. 23946353 PMC3793051

[koag087-B12] Boukas L, Razi A, Bjornsson HT, and Hansen KD. 2022 Oct 17. Natural selection acts on epigenetic marks [preprint]. bioRxiv:2020.07.04.187880. 10.1101/2020.07.04.187880.

[koag087-B13] Box GEP . 1976. Science and statistics. J Am Stat Assoc. 71:791–799. 10.1080/01621459.1976.10480949.

[koag087-B14] Brandes N, Goldman G, Wang CH, Ye CJ, Ntranos V. 2023. Genome-wide prediction of disease variant effects with a deep protein language model. Nat Genet. 55:1512–1522. 10.1038/s41588-023-01465-0. 37563329 PMC10484790

[koag087-B15] Castelló MJ, Carrasco JL, Vera P. 2010. DNA-binding protein phosphatase AtDBP1 mediates susceptibility to two potyviruses in Arabidopsis. Plant Physiol. 153:1521–1525. 10.1104/pp.110.158923. 20508138 PMC2923898

[koag087-B16] Chen W-H et al 2022. Regulatory network for FOREVER YOUNG FLOWER-like genes in regulating Arabidopsis flower senescence and abscission. Commun Biol. 5:662. 10.1038/s42003-022-03629-w. 35790878 PMC9256709

[koag087-B17] Chien P-S, Chen P-H, Lee C-R, Chiou T-J. 2023. Transcriptome-wide association study coupled with eQTL analysis reveals the genetic connection between gene expression and flowering time in Arabidopsis. J Exp Bot. 74:5653–5666. 10.1093/jxb/erad262. 37419660

[koag087-B18] Chizzola R . 2013. Regular monoterpenes and sesquiterpenes (essential oils). In: Ramawat K, Mérillon JM, editors. Natural products. Springer. p. 2973–3008. 10.1007/978-3-642-22144-6_130.

[koag087-B19] Choi K et al 2011. The FRIGIDA complex activates transcription of FLC, a strong flowering repressor in Arabidopsis, by recruiting chromatin modification factors. Plant Cell. 23:289–303. 10.1105/tpc.110.075911. 21282526 PMC3051252

[koag087-B20] Cingolani P et al 2012. A program for annotating and predicting the effects of single nucleotide polymorphisms, SnpEff: SNPs in the genome of Drosophila melanogaster strain w1118; iso-2; iso-3. Fly (Austin). 6:80–92. 10.4161/fly.19695. 22728672 PMC3679285

[koag087-B21] Claessens A, Affara M, Assefa SA, Kwiatkowski DP, Conway DJ. 2017. Culture adaptation of malaria parasites selects for convergent loss-of-function mutants. Sci Rep. 7:41303. 10.1038/srep41303. 28117431 PMC5259787

[koag087-B22] Clarke JH, Dean C. 1994. Mapping FRI, a locus controlling flowering time and vernalization response in Arabidopsis thaliana. Mol Gen Genet. 242:81–89. 10.1007/BF00277351. 7904045

[koag087-B23] Cortinovis G et al 2024. Adaptive gene loss in the common bean pan-genome during range expansion and domestication. Nat Commun. 15:6698. 10.1038/s41467-024-51032-2. 39107305 PMC11303546

[koag087-B24] Deal RB, Henikoff S. 2011. Histone variants and modifications in plant gene regulation. Curr Opin Plant Biol. 14:116–122. 10.1016/j.pbi.2010.11.005. 21159547 PMC3093162

[koag087-B25] Deng W et al 2011. FLOWERING LOCUS C (FLC) regulates development pathways throughout the life cycle of Arabidopsis. Proc Natl Acad Sci U S A. 108:6680–6685. 10.1073/pnas.1103175108. 21464308 PMC3081018

[koag087-B26] Desaint H et al 2024. Reshaping the primary cell wall: dual effects on plant resistance to Ralstonia solanacearum and heat stress response. Mol Plant Microbe Interact. 37:619–634. 10.1094/MPMI-05-24-0059-R. 38904979

[koag087-B27] Eisen MB, Spellman PT, Brown PO, Botstein D. 1998. Cluster analysis and display of genome-wide expression patterns. Proc Natl Acad Sci U S A. 95:14863–14868. 10.1073/pnas.95.25.14863. 9843981 PMC24541

[koag087-B28] Emdin CA et al 2018. Analysis of predicted loss-of-function variants in UK Biobank identifies variants protective for disease. Nat Commun. 9:1613. 10.1038/s41467-018-03911-8. 29691411 PMC5915445

[koag087-B29] Flannick J et al 2014. Loss-of-function mutations in SLC30A8 protect against type 2 diabetes. Nat Genet. 46:357–363. 10.1038/ng.2915. 24584071 PMC4051628

[koag087-B30] Gao W et al 2021. ZjSEP3 modulates flowering time by regulating the LHY promoter. BMC Plant Biol. 21:527. 10.1186/s12870-021-03305-x. 34763664 PMC8582215

[koag087-B31] Gazzani S, Gendall AR, Lister C, Dean C. 2003. Analysis of the molecular basis of flowering time variation in Arabidopsis accessions. Plant Physiol. 132:1107–1114. 10.1104/pp.103.021212. 12805638 PMC167048

[koag087-B32] Ge SX, Jung D, Yao R. 2020. ShinyGO: a graphical gene-set enrichment tool for animals and plants. Bioinformatics. 36:2628–2629. 10.1093/bioinformatics/btz931. 31882993 PMC7178415

[koag087-B33] Giolai M, Laine A-L. 2024. A trade-off between investment in molecular defense repertoires and growth in plants. Science. 386:677–680. 10.1126/science.adn2779. 39509497

[koag087-B34] Göktay M, Fulgione A, Hancock AM. 2021. A new catalog of structural variants in 1,301 A. thaliana lines from Africa, Eurasia, and North America reveals a signature of balancing selection at defense response genes. Mol Biol Evol. 38:1498–1511. 10.1093/molbev/msaa309. 33247723 PMC8042739

[koag087-B35] Gomez MA et al 2019. Simultaneous CRISPR/Cas9-mediated editing of cassava eIF4E isoforms nCBP-1 and nCBP-2 reduces cassava brown streak disease symptom severity and incidence. Plant Biotechnol J. 17:421–434. 10.1111/pbi.12987. 30019807 PMC6335076

[koag087-B36] Hsu H-F, Huang C-H, Chou L-T, Yang C-H. 2003. Ectopic expression of an orchid (Oncidium Gower Ramsey) AGL6-like gene promotes flowering by activating flowering time genes in Arabidopsis thaliana. Plant Cell Physiol. 44:783–794. 10.1093/pcp/pcg099. 12941870

[koag087-B37] Ivarsdottir EV et al 2024. Gene-based burden tests of rare germline variants identify six cancer susceptibility genes. Nat Genet. 56:2422–2433. 10.1038/s41588-024-01966-6. 39472694

[koag087-B38] Jambunathan N, Siani JM, McNellis TW. 2001. A humidity-sensitive Arabidopsis copine mutant exhibits precocious cell death and increased disease resistance. Plant Cell. 13:2225–2240. 10.1105/tpc.010226. 11595798 PMC139155

[koag087-B39] Jamge B et al 2023. Histone variants shape chromatin states in Arabidopsis. eLife. 12:RP87714. 10.7554/elife.87714.2. 37467143 PMC10393023

[koag087-B40] Johanson U et al 2000. Molecular analysis of FRIGIDA, a major determinant of natural variation in Arabidopsis flowering time. Science. 290:344–347. 10.1126/science.290.5490.344. 11030654

[koag087-B41] Jørgensen K et al 2005. Cassava plants with a depleted cyanogenic glucoside content in leaves and tubers. Distribution of cyanogenic glucosides, their site of synthesis and transport, and blockage of the biosynthesis by RNA interference technology. Plant Physiol. 139:363–374. 10.1104/pp.105.065904. 16126856 PMC1203385

[koag087-B42] Kang HM et al 2010. Variance component model to account for sample structure in genome-wide association studies. Nat Genet. 42:348–354. 10.1038/ng.548. 20208533 PMC3092069

[koag087-B43] Kang M et al 2023. The pan-genome and local adaptation of Arabidopsis thaliana. Nat Commun. 14:6259. 10.1038/s41467-023-42029-4. PMC10558531 37802986

[koag087-B44] Katz E et al 2022. Genetic variation underlying differential ammonium and nitrate responses in Arabidopsis thaliana. Plant Cell. 34:4696–4713. 10.1093/plcell/koac279. 36130068 PMC9709984

[koag087-B45] Kawakatsu T et al 2016. Epigenomic diversity in a global collection of Arabidopsis thaliana accessions. Cell. 166:492–505. 10.1016/j.cell.2016.06.044. 27419873 PMC5172462

[koag087-B46] Kim JJ et al 2012. The microRNA156-SQUAMOSA PROMOTER BINDING PROTEIN-LIKE3 module regulates ambient temperature-responsive flowering via FLOWERING LOCUS T in Arabidopsis. Plant Physiol. 159:461–478. 10.1104/pp.111.192369. 22427344 PMC3375978

[koag087-B47] Kliebenstein D . 2009. Quantitative genomics: analyzing intraspecific variation using global gene expression polymorphisms or eQTLs. Annu Rev Plant Biol. 60:93–114. 10.1146/annurev.arplant.043008.092114. 19012536

[koag087-B48] Klim J, Zielenkiewicz U, Kaczanowski S. 2024. Loss-of-function mutations are main drivers of adaptations during short-term evolution. Sci Rep. 14:7128. 10.1038/s41598-024-57694-8. 38532077 PMC10965932

[koag087-B49] Koprulu M et al 2022. Identification of rare loss-of-function genetic variation regulating body fat distribution. J Clin Endocrinol Metab. 107:1065–1077. 10.1210/clinem/dgab877. 34875679 PMC8947777

[koag087-B50] Kvitek DJ, Sherlock G. 2013. Whole genome, whole population sequencing reveals that loss of signaling networks is the major adaptive strategy in a constant environment. PLoS Genet. 9:e1003972. 10.1371/journal.pgen.1003972. 24278038 PMC3836717

[koag087-B51] Lee G et al 2024. A large-effect fitness trade-off across environments is explained by a single mutation affecting cold acclimation. Proc Natl Acad Sci U S A. 121:e2317461121. 10.1073/pnas.2317461121. 38289961 PMC10861903

[koag087-B52] Lee H-T, Oh S, Ro DH, Yoo H, Kwon Y-W. 2020. The key role of DNA methylation and histone acetylation in epigenetics of atherosclerosis. J Lipid Atheroscler. 9:419–434. 10.12997/jla.2020.9.3.419. 33024734 PMC7521974

[koag087-B53] Lee S, Abecasis GR, Boehnke M, Lin X. 2014. Rare-variant association analysis: study designs and statistical tests. Am J Hum Genet. 95:5–23. 10.1016/j.ajhg.2014.06.009. 24995866 PMC4085641

[koag087-B54] Li S-X, Liu Y, Zhang Y-M, Chen J-Q, Shao Z-Q. 2025. Convergent reduction of immune receptor repertoires during plant adaptation to diverse special lifestyles and habitats. Nat Plants. 11:1–15. 10.1038/s41477-025-01911-3. 39821112

[koag087-B55] Liu D-X et al 2021. Calling large indels in 1047 Arabidopsis with IndelEnsembler. Nucleic Acids Res. 49:10879–10894. 10.1093/nar/gkab904. 34643730 PMC8565333

[koag087-B56] Liu J et al 2024. The goat pan-genome reveals patterns of gene loss during domestication. J Anim Sci Biotechnol. 15:132. 10.1186/s40104-024-01092-7. 39367490 PMC11453020

[koag087-B57] Liu R et al 1996. Homozygous defect in HIV-1 coreceptor accounts for resistance of some multiply-exposed individuals to HIV-1 infection. Cell. 86:367–377. 10.1016/S0092-8674(00)80110-5. 8756719

[koag087-B58] Liu Y et al 2018. PCSD: a plant chromatin state database. Nucleic Acids Res. 46:D1157–D1167. 10.1093/nar/gkx919. 29040761 PMC5753246

[koag087-B59] Lloyd J, Meinke D. 2012. A comprehensive dataset of genes with a loss-of-function mutant phenotype in Arabidopsis. Plant Physiol. 158:1115–1129. 10.1104/pp.111.192393. 22247268 PMC3291275

[koag087-B60] Lu F et al 2008. Comparative analysis of JmjC domain-containing proteins reveals the potential histone demethylases in Arabidopsis and rice. J Integr Plant Biol. 50:886–896. 10.1111/j.1744-7909.2008.00692.x. 18713399

[koag087-B61] Lu Z et al 2019. The prevalence, evolution and chromatin signatures of plant regulatory elements. Nat Plants. 5:1250–1259. 10.1038/s41477-019-0548-z. 31740772

[koag087-B62] MacArthur DG et al 2012. A systematic survey of loss-of-function variants in human protein-coding genes. Science. 335:823–828. 10.1126/science.1215040. 22344438 PMC3299548

[koag087-B63] Mandel MA, Yanofsky MF. 1998. The Arabidopsis AGL 9 MADS box gene is expressed in young flower primordia. Sex Plant Reprod. 11:22–28. 10.1007/s004970050116.

[koag087-B64] Manolio TA . 2010. Genomewide association studies and assessment of the risk of disease. N Engl J Med. 363:166–176. 10.1056/NEJMra0905980. 20647212

[koag087-B65] Marbach D et al 2016. Tissue-specific regulatory circuits reveal variable modular perturbations across complex diseases. Nat Methods. 13:366–370. 10.1038/nmeth.3799. 26950747 PMC4967716

[koag087-B66] McCaw ZR, Lane JM, Saxena R, Redline S, Lin X. 2020. Operating characteristics of the rank-based inverse normal transformation for quantitative trait analysis in genome-wide association studies. Biometrics. 76:1262–1272. 10.1111/biom.13214. 31883270 PMC8643141

[koag087-B67] McDonald JH, Kreitman M. 1991. Adaptive protein evolution at the Adh locus in Drosophila. Nature. 351:652–654. 10.1038/351652a0. 1904993

[koag087-B68] Mergner J et al 2020. Mass-spectrometry-based draft of the Arabidopsis proteome. Nature. 579:409–414. 10.1038/s41586-020-2094-2. 32188942

[koag087-B69] Minikel EV et al 2020. Evaluating drug targets through human loss-of-function genetic variation. Nature. 581:459–464. 10.1038/s41586-020-2267-z. 32461653 PMC7272226

[koag087-B70] Monroe JG et al 2016. Adaptation to warmer climates by parallel functional evolution of CBF genes in Arabidopsis thaliana. Mol Ecol. 25:3632–3644. 10.1111/mec.13711. 27247130

[koag087-B71] Monroe JG et al 2022. Mutation bias reflects natural selection in Arabidopsis thaliana. Nature. 602:101–105. 10.1038/s41586-021-04269-6. 35022609 PMC8810380

[koag087-B72] Monroe JG, McKay JK, Weigel D, Flood PJ. 2021. The population genomics of adaptive loss of function. Heredity (Edinb). 126:383–395. 10.1038/s41437-021-00403-2. 33574599 PMC7878030

[koag087-B73] Murray AW . 2020. Can gene-inactivating mutations lead to evolutionary novelty? Curr Biol. 30:R465–R471. 10.1016/j.cub.2020.03.072. 32428483

[koag087-B74] Neville MDC, et al 2024. Sperm sequencing reveals extensive positive selection in the male germline. Nature. 647:421–428. 10.1101/2024.10.30.24316414. PMC12611766 41062690

[koag087-B75] Noma K, Allis CD, Grewal SI. 2001. Transitions in distinct histone H3 methylation patterns at the heterochromatin domain boundaries. Science. 293:1150–1155. 10.1126/science.1064150. 11498594

[koag087-B76] Olson MV . 1999. When less is more: gene loss as an engine of evolutionary change. Am J Hum Genet. 64:18–23. 10.1086/302219. 9915938 PMC1377697

[koag087-B77] Orr HA . 2005. The genetic theory of adaptation: a brief history. Nat Rev Genet. 6:119–127. 10.1038/nrg1523. 15716908

[koag087-B78] Pan M-R, Hsu M-C, Chen L-T, Hung W-C. 2018. Orchestration of H3K27 methylation: mechanisms and therapeutic implication. Cell Mol Life Sci. 75:209–223. 10.1007/s00018-017-2596-8. 28717873 PMC5756243

[koag087-B79] Paparella C, Savatin DV, Marti L, De Lorenzo G, Ferrari S. 2014. The Arabidopsis LYSIN MOTIF-CONTAINING RECEPTOR-LIKE KINASE3 regulates the cross talk between immunity and abscisic acid responses. Plant Physiol. 165:262–276. 10.1104/pp.113.233759. 24639336 PMC4012585

[koag087-B80] Parenicová L et al 2003. Molecular and phylogenetic analyses of the complete MADS-box transcription factor family in Arabidopsis: new openings to the MADS world: new openings to the MADS world. Plant Cell. 15:1538–1551. 10.1105/tpc.011544. 12837945 PMC165399

[koag087-B81] Pérez-Ruiz RV et al 2015. XAANTAL2 (AGL14) is an important component of the complex gene regulatory network that underlies Arabidopsis shoot apical meristem transitions. Mol Plant. 8:796–813. 10.1016/j.molp.2015.01.017. 25636918

[koag087-B82] Poulicard N et al 2024. Repeated loss of the ability of a wild pepper disease resistance gene to function at high temperatures suggests that thermoresistance is a costly trait. New Phytol. 241:845–860. 10.1111/nph.19371. 37920100

[koag087-B83] Povysil G et al 2019. Rare-variant collapsing analyses for complex traits: guidelines and applications. Nat Rev Genet. 20:747–759. 10.1038/s41576-019-0177-4. 31605095

[koag087-B84] Quiroz D et al 2024. H3k4me1 recruits DNA repair proteins in plants. Plant Cell. 36:2410–2426. 10.1093/plcell/koae089. 38531669 PMC11132887

[koag087-B85] Rausell A et al 2020. Common homozygosity for predicted loss-of-function variants reveals both redundant and advantageous effects of dispensable human genes. Proc Natl Acad Sci U S A. 117:13626–13636. 10.1073/pnas.1917993117. 32487729 PMC7306792

[koag087-B86] Risk JM et al 2010. FRIGIDA and related proteins have a conserved central domain and family specific N- and C- terminal regions that are functionally important. Plant Mol Biol. 73:493–505. 10.1007/s11103-010-9635-2. 20405310

[koag087-B87] Roth SY, Denu JM, Allis CD. 2001. Histone acetyltransferases. Annu Rev Biochem. 70:81–120. 10.1146/annurev.biochem.70.1.81. 11395403

[koag087-B88] Sánchez-Serna G et al 2025. Less, but more: new insights from appendicularians on chordate Fgf evolution and the divergence of tunicate lifestyles. Mol Biol Evol. 42:msae260. 10.1093/molbev/msae260. 39686543 PMC11733497

[koag087-B89] Schotta G et al 2002. Central role of Drosophila SU(VAR)3-9 in histone H3-K9 methylation and heterochromatic gene silencing. EMBO J. 21:1121–1131. 10.1093/emboj/21.5.1121. 11867540 PMC125909

[koag087-B90] Sharma V et al 2018. A genomics approach reveals insights into the importance of gene losses for mammalian adaptations. Nat Commun. 9:1215. 10.1038/s41467-018-03667-1. 29572503 PMC5865188

[koag087-B91] Smith JM . 1970. Natural selection and the concept of a protein space. Nature. 225:563–564. 10.1038/225563a0. 5411867

[koag087-B92] Sonawane AR et al 2017. Understanding tissue-specific gene regulation. Cell Rep. 21:1077–1088. 10.1016/j.celrep.2017.10.001. 29069589 PMC5828531

[koag087-B93] Spence JP et al 2026. Specificity, length and luck drive gene rankings in association studies. Nature. 649:918–925. 10.1038/s41586-025-09703-7. 41193809 PMC12823407

[koag087-B94] Spielmeyer W, Ellis MH, Chandler PM. 2002. Semidwarf (sd-1), “green revolution” rice, contains a defective gibberellin 20-oxidase gene. Proc Natl Acad Sci U S A. 99:9043–9048. 10.1073/pnas.132266399. 12077303 PMC124420

[koag087-B95] Uffelmann E et al 2021. Genome-wide association studies. Nat Rev Methods Primers. 1:1–21. 10.1038/s43586-021-00056-9.

[koag087-B96] Wang F et al 2015. Functional analysis of the Arabidopsis TETRASPANIN gene family in plant growth and development. Plant Physiol. 169:2200–2214. 10.1104/pp.15.01310. 26417009 PMC4634101

[koag087-B97] Wang F, Vandepoele K, Van Lijsebettens M. 2012. Tetraspanin genes in plants. Plant Sci. 190:9–15. 10.1016/j.plantsci.2012.03.005. 22608515

[koag087-B98] Wang W et al 2018. Genomic variation in 3,010 diverse accessions of Asian cultivated rice. Nature. 557:43–49. 10.1038/s41586-018-0063-9. 29695866 PMC6784863

[koag087-B99] Wang Y et al 2019. The IMMUNE-ASSOCIATED NUCLEOTIDE-BINDING 9 protein is a regulator of basal immunity in Arabidopsis thaliana. Mol Plant Microbe Interact. 32:65–75. 10.1094/MPMI-03-18-0062-R. 29958083

[koag087-B100] Wisecaver JH et al 2017. A global coexpression network approach for connecting genes to specialized metabolic pathways in plants. Plant Cell. 29:944–959. 10.1105/tpc.17.00009. 28408660 PMC5466033

[koag087-B101] Wong A, Rodrigue N, Kassen R. 2012. Genomics of adaptation during experimental evolution of the opportunistic pathogen Pseudomonas aeruginosa. PLoS Genet. 8:e1002928. 10.1371/journal.pgen.1002928. 23028345 PMC3441735

[koag087-B102] Xia S et al 2009. Negative regulation of systemic acquired resistance by replication factor C subunit3 in Arabidopsis. Plant Physiol. 150:2009–2017. 10.1104/pp.109.138321. 19482917 PMC2719121

[koag087-B103] Xu M et al 2016. Developmental functions of miR156-regulated SQUAMOSA PROMOTER BINDING PROTEIN-LIKE (SPL) genes in Arabidopsis thaliana. PLoS Genet. 12:e1006263. 10.1371/journal.pgen.1006263. 27541584 PMC4991793

[koag087-B104] Xu Y-C et al 2019. Adaptation and phenotypic diversification in Arabidopsis through loss-of-function mutations in protein-coding genes. Plant Cell. 31:1012–1025. 10.1105/tpc.18.00791. 30886128 PMC6533021

[koag087-B105] Yang H et al 2012a. A companion cell-dominant and developmentally regulated H3K4 demethylase controls flowering time in Arabidopsis via the repression of FLC expression. PLoS Genet. 8:e1002664. 10.1371/journal.pgen.1002664. 22536163 PMC3334889

[koag087-B106] Yang H et al 2012b. Overexpression of a histone H3K4 demethylase, JMJ15, accelerates flowering time in Arabidopsis. Plant Cell Rep. 31:1297–1308. 10.1007/s00299-012-1249-5. 22555401

[koag087-B107] Yang W, Jiang D, Jiang J, He Y. 2010. A plant-specific histone H3 lysine 4 demethylase represses the floral transition in Arabidopsis. Plant J. 62:663–673. 10.1111/j.1365-313X.2010.04182.x. 20202164

[koag087-B108] Yoo SK et al 2005. CONSTANS activates SUPPRESSOR OF OVEREXPRESSION OF CONSTANS 1 through FLOWERING LOCUS T to promote flowering in Arabidopsis. Plant Physiol. 139:770–778. 10.1104/pp.105.066928. 16183837 PMC1255994

[koag087-B109] Yu H, Goh CJ. 2000. Identification and characterization of three orchid MADS-box genes of the AP1/AGL9 subfamily during floral transition. Plant Physiol. 123:1325–1336. 10.1104/pp.123.4.1325. 10938351 PMC59091

[koag087-B110] Zhang B et al 2025. Adaptive loss of shortwave-sensitive opsins during cartilaginous fish evolution. Nat Commun. 16:7684. 10.1038/s41467-025-62544-w. 40825943 PMC12361510

[koag087-B111] Zmienko A et al 2020. AthCNV: a map of DNA copy number variations in the Arabidopsis genome. Plant Cell. 32:1797–1819. 10.1105/tpc.19.00640. 32265262 PMC7268809

