# Supplementary material for: Functional insights into dispensable genes using genome-wide loss-of-function burden tests in Arabidopsis
Source: Plant Cell. 2026 Mar 27;38(4):koag087. doi: 10.1093/plcell/koag087 (PMC13089410; doi:10.1093/plcell/koag087)
Supplement: koag087_Supplementary_Data [file koag087_supplementary_data.zip › Supplementary_Data.pdf]

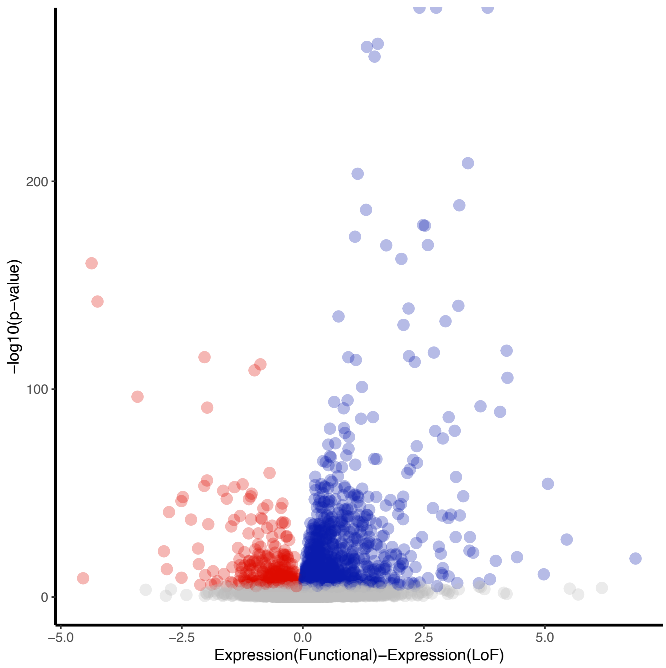

Figure S1. Predicted LoF (pLoF) Alleles Display Generally Lower Expression Level Than Their Functional Counterparts. Each point represents a t-test between the expression level of pLoF alleles and functional alleles of a gene. Blue: functional alleles have higher expression than pLoF alleles; red: pLoF alleles have higher expression than functional alleles; grey: not significant in t-test after multiple testing correction.

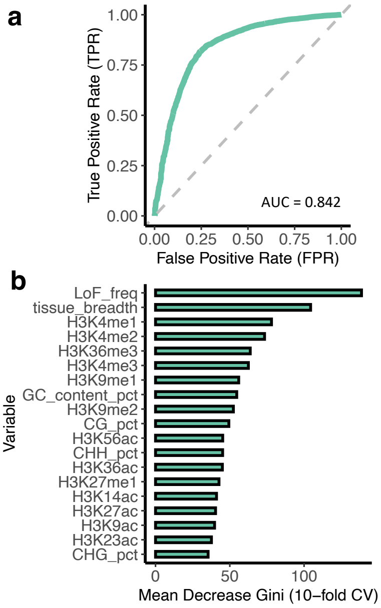

Figure S2. Random Forest Model of Pan-genome Gene Dispensability Prediction Categorizing Softcore Genes as Indispensable. a) The ROC curve of the predictive model using Random Forest (RF) categorizing softcore genes as indispensable. An AUC-ROC value of ~0.5 is equivalent to random guessing, while an AUC-ROC of 1 indicates perfect predictions. The diagonal dashed line shows the expected performance of a model based on random guessing. Curves closer to the upper left corner of the chart represent a better predictive performance than curves that are closer to the diagonal dashed line. b) The Mean Decrease Gini of all the predictors in the RF model. Higher Gini score signifies higher importance of the variable to the predictive model.

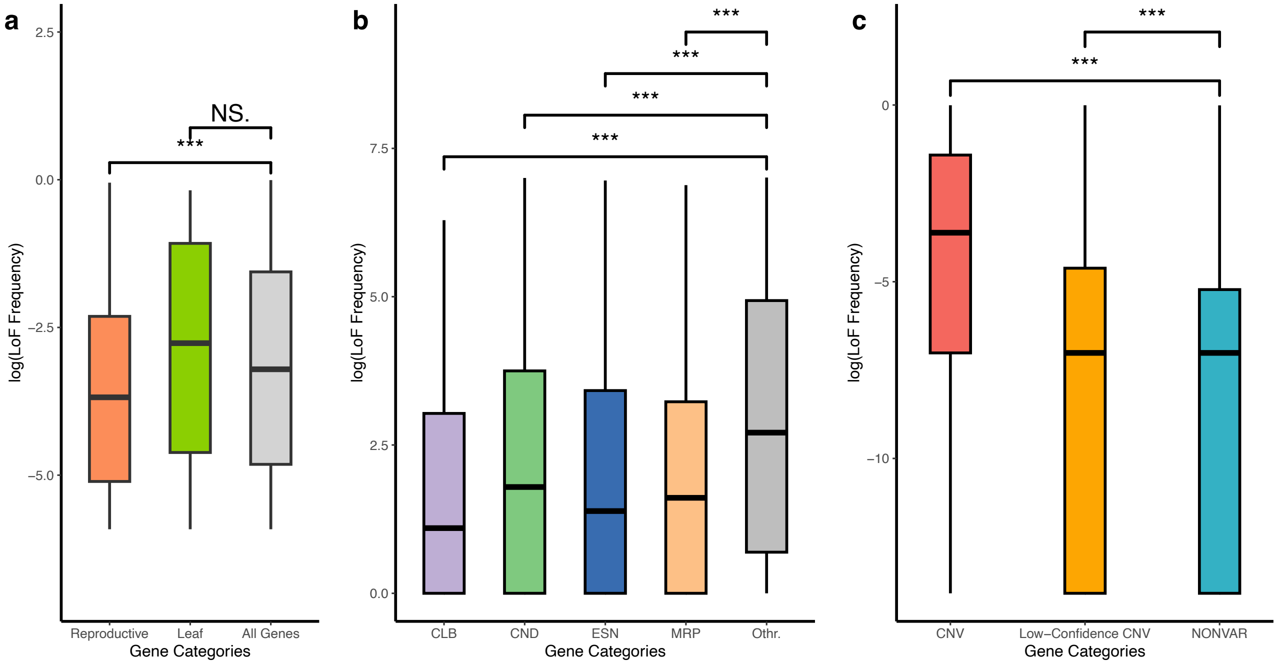

Figure S3. Loss-of-function Frequency in Different Gene Categories. a) Boxplots of gene LoF frequency of genes highly expressed in leaf tissues, reproductive tissues, and all genes, respectively. Genes highly expressed in one type of tissue is defined as at least twice average transcript level than other tissue types. Leaf tissues include vascular leaf, cauline leaf, and rosette leaf. Reproductive tissues include fruit, seed, embryo, flower, silique. Genes highly expressed in reproductive tissues display significantly lower LoF frequency. b) Boxplots of LoF frequency of genes according to their essentiality categories based on Lloyd and Meinke 2012. Genes are categorized based on their mutant phenotypes. CLB: cellular and biochemical; CND: conditional; ESN: essential; MRP: morphological; Othr.: other genes. c) Boxplots of gene LoF frequency of Copy Number Variation (CNV) genes and NONVAR-genes (genes that did not overlap with any CNVs). NONVAR-genes show significantly lower LoF frequency than CNV-genes. For all panels: The center line indicates the median; boxes represent the interquartile range (25th–75th percentiles); whiskers extend to 1.5× the interquartile range. Statistical significance was assessed using a two-sided Student's t-test. *** indicates $P < 0.001$; NS indicates not significant.

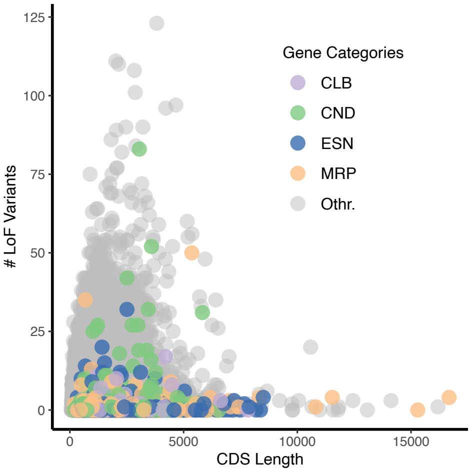

Figure S4. Loss-of-function Variant Number in Relation to Gene Length. A positive correlation was observed between gene CDS length and the number of LoF variants accumulated. Data points were colored by gene categories based on their mutant phenotypes from Lloyd and Meinke 2012. CLB: cellular and biochemical; CND: conditional; ESN: essential; MRP: morphological; Othr.: other genes.

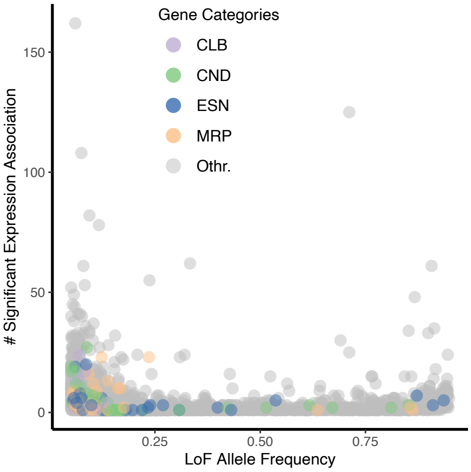

Figure S5. Rare Alleles Show More Expression Associations in LoF Burden Tests. Data points were colored by gene categories based on their mutant phenotypes from Lloyd and Meinke 2012. CLB: cellular and biochemical; CND: conditional; ESN: essential; MRP: morphological; Othr.: other genes.

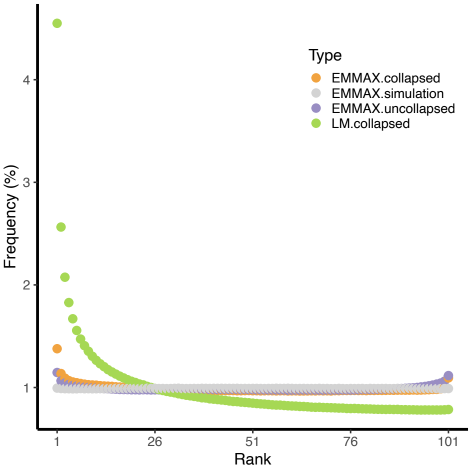

Figure S6. Rank Distribution of LoF-Expression Associations against Simulations Using Different Approaches. Orange: LoF burden tests using EMMAX; Purple: LoF association testing without aggregating LoF burdens; Green: LoF burden tests using a general linear model; Grey: Simulated LoF burden tests using EMMAX by permuting LoF and functional alleles.

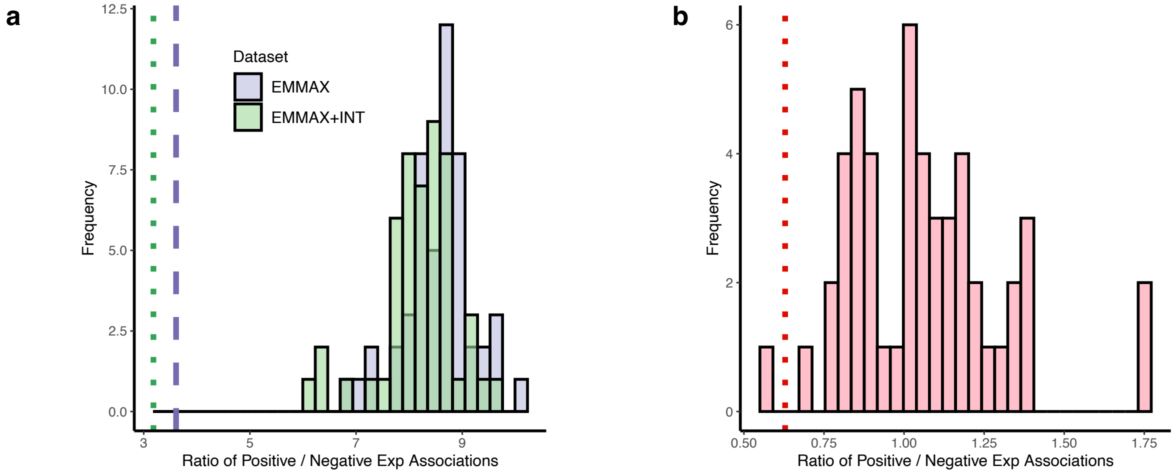

Figure S7. Skewed Expression Data Drives Bias Toward Positive LoF-Expression Associations. a) Histogram of positive-to-negative association ratios from 50 simulated genome-wide LoF burden tests, comparing results before and after inverse normal transformation (INT) of expression data. The purple dashed line indicates the observed positive-to-negative association ratio in the empirical dataset (~3.6), while the green dotted line represents the ratio after INT (~3.2). b) Histogram of positive-to-negative association ratios from 50 simulated genome-wide LoF burden tests after removing non-normally distributed expression genes. The red dashed line marks the observed positive-to-negative association ratio in the empirical dataset after filtering (~0.63). Other filtering criteria: 0.05 < LoF allele frequency < 0.95; FDR-corrected $p < 0.05$.

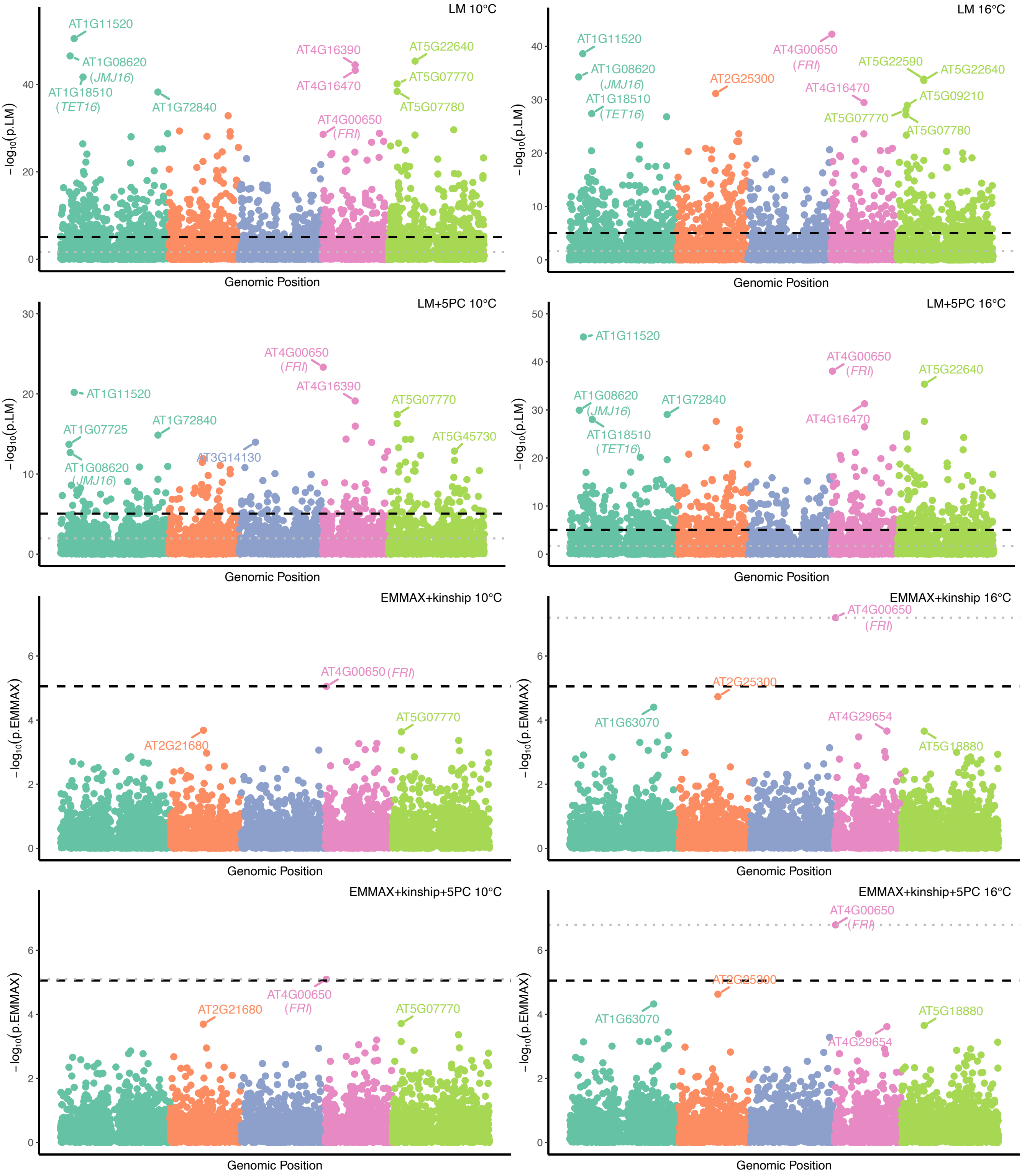

Figure S8. Manhattan Plots of Flowering Time Association under Different Models and Conditions. Genome-wide association results are shown for flowering time at 10 °C (left panels) and 16 °C (right panels). Results are presented for four models: a simple linear model (LM; top row), LM with the first five principal components included as covariates (second row), EMMAX with a kinship matrix (third row), and EMMAX with kinship plus five PCs (bottom row). The x-axis indicates genomic position along the five Arabidopsis chromosomes, and the y-axis shows $-\log_{10}(p)$ values. Dashed black lines represent the Bonferroni significance threshold, and dotted grey lines represent the largest significant FDR-adjusted p-value used for each method.

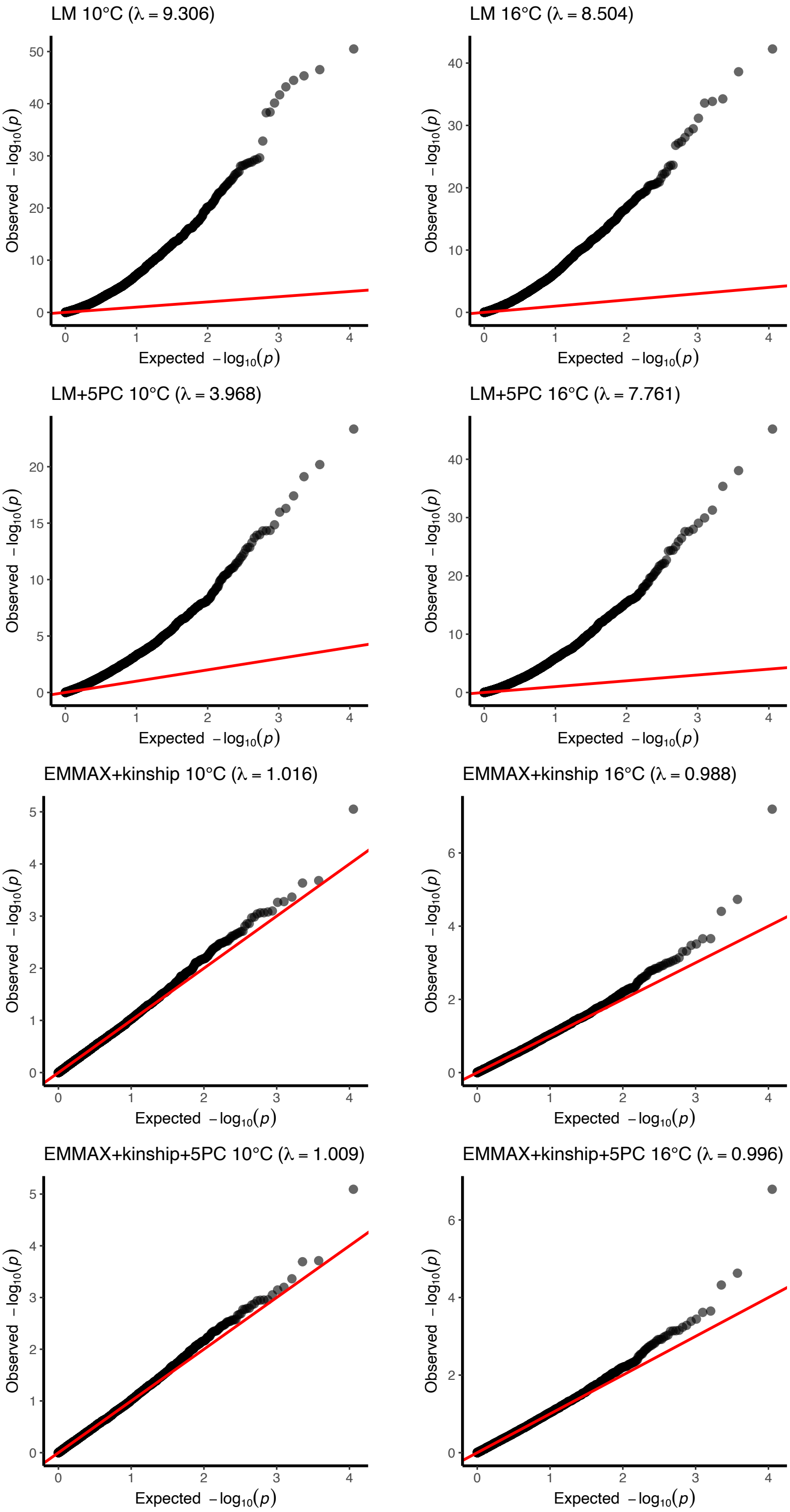

Figure S9. Quantile–Quantile (QQ) Plots of Flowering Time Association under Different Models and Conditions. Observed versus expected $-\log_{10}(p)$ values are shown for association tests at 10 °C (left panels) and 16 °C (right panels). Results are presented for four models: a simple linear model (LM; top row), LM with the first five principal components included as covariates (second row), EMMAX with a kinship matrix (third row), and EMMAX with kinship plus five PCs (bottom row). The genomic inflation factor (λ) is reported for each analysis. The red line indicates the expected null distribution, and deviation above the line indicates inflation of association statistics.
